# Exploring the mediating role of calcium homeostasis in the association between diabetes mellitus, glycemic traits, and vascular and valvular calcifications: a comprehensive Mendelian randomization analysis

**DOI:** 10.1186/s13098-024-01383-z

**Published:** 2024-06-22

**Authors:** Xian-Guan Zhu, Gui-Qin Liu, Ya-Ping Peng, Li-Ling Zhang, Xian-Jin Wang, Liang-Chuan Chen, Yuan-Xi Zheng, Rui Qiao, Xue-Jun Xiang, Xian-He Lin

**Affiliations:** 1 Department of Cardiology, Anqing Municipal Hospital, Anqing, 246000 Anhui China; 2 Graduate School, Wannan Medical College, Wuhu, 241002 Anhui China; 3 Department of Cardiology, The First Affiliated Hospital of Anhui Medical University, Hefei, 230000 Anhui China

**Keywords:** Mendelian randomization, Diabetes mellitus, Glycemic traits, Calcium homeostasis, Vascular and valvular calcifications, Mediation analysis

## Abstract

**Background:**

The interplay between diabetes mellitus (DM), glycemic traits, and vascular and valvular calcifications is intricate and multifactorial. Exploring potential mediators may illuminate underlying pathways and identify novel therapeutic targets.

**Methods:**

We utilized univariable and multivariable Mendelian randomization (MR) analyses to investigate associations and mediation effects. Additionally, the multivariable MR analyses incorporated cardiometabolic risk factors, allowing us to account for potential confounders.

**Results:**

Type 2 diabetes mellitus (T2DM) and glycated hemoglobin (HbA1c) were positively associated with both coronary artery calcification (CAC) and calcific aortic valvular stenosis (CAVS). However, fasting glucose (FG) was only linked to CAVS and showed no association with CAC. Additionally, CAVS demonstrated a causal effect on FG. Calcium levels partially mediated the impact of T2DM on both types of calcifications. Specifically, serum calcium was positively associated with both CAC and CAVS. The mediation effects of calcium levels on the impact of T2DM on CAC and CAVS were 6.063% and 3.939%, respectively. The associations between T2DM and HbA1c with calcifications were influenced by body mass index (BMI) and smoking status. However, these associations were generally reduced after adjusting for hypertension.

**Conclusion:**

Our findings suggest a genetically supported causal relationship between DM, glycemic traits, and vascular and valvular calcifications, with serum calcium playing a critical mediating role.

**Supplementary Information:**

The online version contains supplementary material available at 10.1186/s13098-024-01383-z.

## Introduction

Diabetes mellitus (DM) has become an increasingly prevalent and potentially devastating medical condition, now recognized as a significant global health concern [1]. The morbidity and mortality associated with DM are primarily due to its complications. These complications are traditionally categorized into macrovascular disease, which includes cardiovascular disease (CVD), and microvascular disease, which encompasses retinopathy and neuropathy [2]. Epidemiological studies have established that vascular and valvular calcifications are independent and robust predictors of CVD and major adverse cardiac events (MACE) [3, 4].

Vascular and valvular calcifications primarily manifest as coronary artery calcification (CAC) and calcific aortic valvular stenosis (CAVS). Interestingly, studies show that up to 50% of patients with CAVS have concomitant coronary artery disease. This strong association is believed to be due to the shared pathobiology and risk factors, which could extend to metabolic diseases like diabetes [5]. Observational studies show that these conditions involve pathological deposits of calcium phosphate salts in the cardiovascular system, particularly in the coronary arteries and heart valves [6]. These calcifications are not merely passive indicators of disease but actively contribute to the pathology of cardiovascular structures. In the coronary arteries, calcifications diminish elasticity, leading to increased systolic blood pressure and left ventricular hypertrophy, which often progresses to heart failure [7]. In cardiac valves, these calcifications cause stiffness and decreased mobility, resulting in valve diseases like aortic stenosis, which severely impair cardiac output and function. Its prevalence increases with age, affecting over 50% of individuals over the age of 85 in high-income countries [8].

Although epidemiological studies have revealed potential associations between these calcifications and metabolic disorders such as DM and altered glycemic traits, the underlying mechanisms driving this association remain complex, multifaceted, and inadequately understood [9, 10]. Additionally, retrospective and prospective analyses have revealed that obesity, smoking, and hypertension are also associated with an increased risk of vascular and valvular calcifications [5, 9]. Moreover, emerging research has pointed to calcium homeostasis as a potential mediator in the progression of cardiovascular calcifications in diabetic individuals [11]. This mediating role is critical, as it suggests that disruptions in calcium metabolic processes could link metabolic diseases to vascular and valvular calcifications. The growing global prevalence of these calcifications and the absence of effective non-surgical treatments underscore the urgent need for therapeutic strategies rooted in a deeper understanding of the disease mechanisms.

Furthermore, although a series of observational studies have demonstrated a positive association between DM and vascular and valvular calcifications, we do not know whether diabetes directly causes these calcifications or if the associations are biased by known confounders, including a range of modifiable risk factors [5], and even unknown confounders that might also lead to cardiovascular calcifications. Therefore, whether there are causal effects of DM and glycemic traits (including glycated hemoglobin [HbA1c], fasting insulin [FI], and fasting glucose [FG]) on vascular and valvular calcifications, as well as the mediating effect of calcium homeostasis, remains unknown.

To dissect these complex associations, we employed a two-sample, two-step, and multivariable Mendelian Randomization (MR) analyses using single nucleotide polymorphisms (SNPs) that are robustly associated with DM and glycemic traits. This methodological framework allows for the evaluation of causal relationships while mitigating confounding factors typically encountered in observational studies [12]. Briefly, our comprehensive analysis included both univariable and multivariable MR assessments to delineate the associations and potential mediation effects of calcium homeostasis in the development of vascular and valvular calcifications. Additionally, we adjusted for relevant cardiometabolic risk factors such as body mass index (BMI), smoking status, and hypertension [5], further refining our understanding of these complex interactions. This study also provides clues for further research direction on therapeutic targets for both types of calcifications.

## Methods

### Study design

**Fig. 1:**
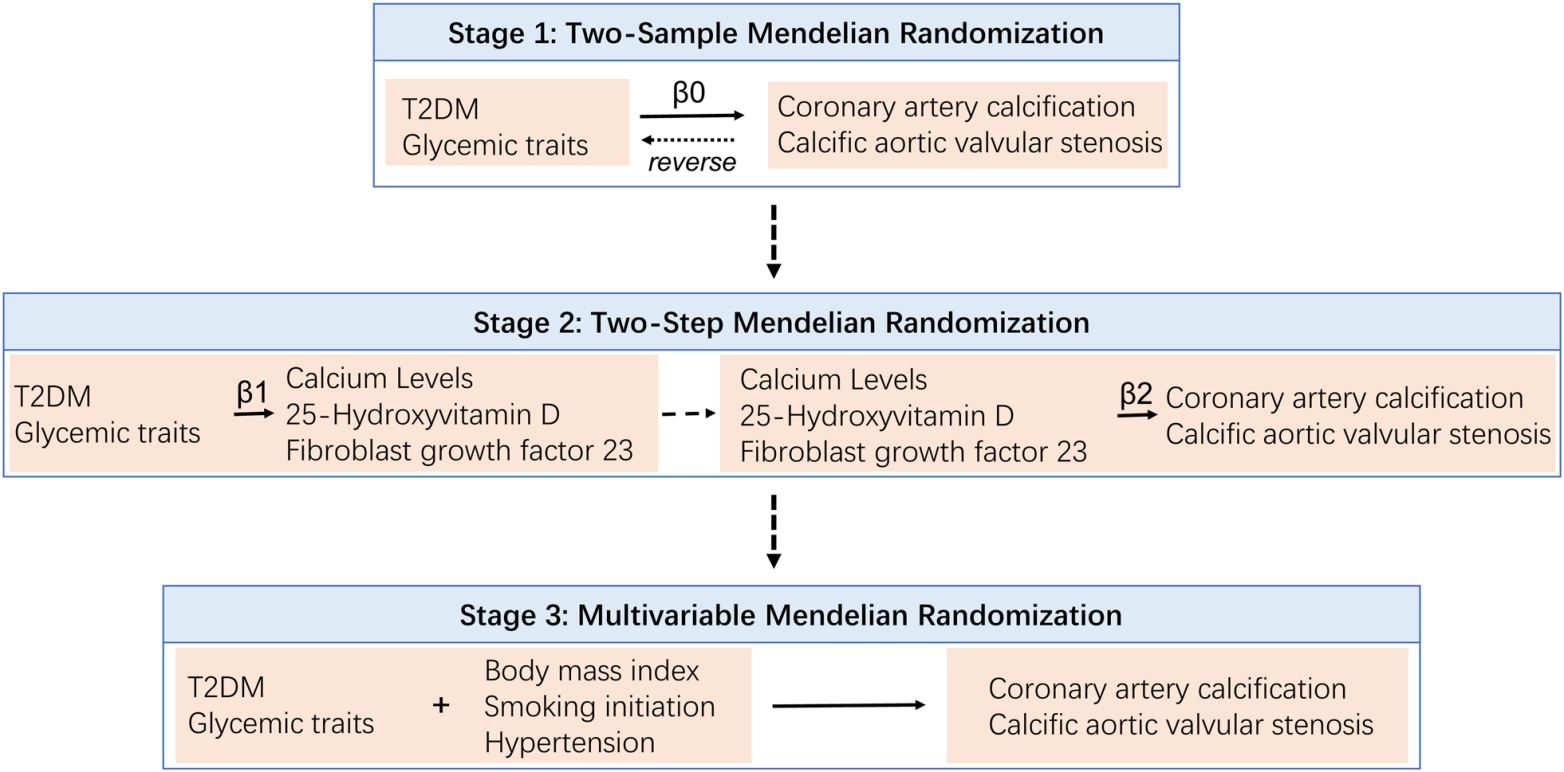
The study's overall workflow. Three stages of analyses designed to meticulously explore the mediating role of calcium homeostasis in the association between diabetes mellitus, glycemic traits, and vascular and valvular calcifications. The detailed description can be found in the main text

Figure 1 illustrates the study design diagram, which comprises three stages of analyses aimed at thoroughly examining the causal relationships between DM, glycemic traits, and vascular and valvular calcifications.

Stage 1: We conducted two-sample MR analyses to assess the total effects of DM and glycemic traits on both types of calcifications, denoted as β0. Additionally, we performed reverse MR analyses to investigate potential reverse causality from these calcifications back to DM and glycemic traits.

Stage 2: Two-sample two-step MR analyses were carried out to identify potential intermediaries in the pathway linking DM and glycemic traits to vascular and valvular calcifications. We quantified the proportion of the mediating effect of DM and glycemic traits on these calcifications through calcium homeostasis, calculated as (β1 * β2)/β0 [13].

Stage 3: We performed multivariable MR analyses to evaluate the independent associations between DM, glycemic traits, and both types of calcifications, accounting for the potential influence of cardiometabolic risk factors such as BMI, smoking initiation, systolic blood pressure, and diastolic blood pressure.

Briefly, our study adopted a comprehensive MR approach, utilizing two-sample, two-step, and multivariable analyses. We used SNPs from genome-wide association studies (GWAS) as instrumental variables (IVs) to estimate causal relationships between DM, glycemic traits, and the risk of vascular and valvular calcifications. For MR analyses to yield valid results, three critical assumptions must be satisfied: (1) the SNPs are associated with the exposure; (2) the SNPs are independent of confounders of the exposure-outcome relationship (the independence assumption); and (3) the SNPs affect the outcome only through the exposure. Informed consent and ethics approval were obtained for each of the original studies [13].

### Exposure GWAS

Initially, we selected type 2 diabetes mellitus (T2DM) from a GWAS dataset comprising 62,892 diabetic patients and 596,424 controls of European ancestry. The diagnosis of T2DM was established using various methods, as described in the primary GWAS publications [14]. Additionally, we selected variants associated with key glycemic traits, including HbA1c levels [15], FI [16], and FG [16], from corresponding GWAS publications.

### Mediator GWAS

Summary statistics for calcium homeostasis, including serum calcium, 25-hydroxyvitamin D (25OHD), and fibroblast growth factor 23 (FGF23), were extracted from three distinct GWASs. For serum calcium, the data were obtained from 400,792 samples within the UK Biobank [17]. The genetic predictors for serum 25OHD were sourced from the largest available GWAS, involving 496,946 participants [18]. Additionally, the summary statistics for FGF23 were obtained from a study comprising 21,758 samples [19].

### Outcome GWAS

Data on CAC were obtained from an open GWAS published by Kavousi M et al. (2023), encompassing a total of 28,655 individuals [20]. The source for this data is available at CVD HugeAmp (https://cvd.hugeamp.org). Additionally, data on CAVS were extracted from FinnGen (https://www.finngen.fi/en), which included 9153 cases and 402,311 controls. Further details can be found on the FinnGen website.

### Cardiometabolic risk factors GWAS

Summary statistics for cardiometabolic risk factors were extracted from three distinct GWASs. These factors include BMI, with a sample size of 532,396 [21]; smoking initiation, with 607,291 participants [22]; and both systolic and diastolic blood pressure, each with 810,865 participants [23].

All summary data utilized in this study are publicly accessible, with detailed information presented in Supplementary Tables S1.

### Selection of IVs

We identified SNPs as IVs using three criteria: significant genome-wide association (*p* < 5e−8), confirmed independent heritability (*r*^*2*^ < 0.001), and the absence of linkage disequilibrium, assessed over a dense 10,000 kb window [24]. The evaluation of weak associations between SNPs and exposures was based on the formula F = R^2^ × (N − 2)/(1 − R^2^). R^2^ was estimated via employing the equation: R^2^ = 2 × MAF × (1 − MAF) × β^2^, where β is the allele effect value and MAF is the effect allele frequency. SNPs with an F-statistic greater than 10 were considered reliable IVs due to their ability to reduce bias associated with weak instruments in MR analyses [25].

### Statistical analysis

All analyses were conducted using R (version 4.3.2). For MR analyses, we employed the "TwoSampleMR" package and identified outliers with the "MR-PRESSO" package [26]. We applied four different MR methods in the univariable MR analyses: inverse variance weighted (IVW), MR-Egger regression, weighted median, and weighted mode. The majority of our statistical evaluations utilized the IVW method, which is recognized for its robustness in identifying causal relationships in univariable MR analyses. For multivariable MR analyses, we used the "MVMR" and "MendelianRandomization" packages [27]. Multivariable MR was employed to investigate the direct causal relationships between T2DM, glycemic traits, and vascular and valvular calcifications. We also applied several multivariable MR techniques—including multivariable MR-IVW, multivariable MR-median, and the least absolute shrinkage and selection operator (LASSO)—to assess direct causality, adjusting for cardiometabolic risk factors. If at least one of these three methods yields a significant result, it is considered that the causal relationship still exists even after multivariable adjustment [28].

To enhance the reliability of our experimental results and comply with the assumptions of MR, we executed an extensive range of sensitivity analyses. These analyses included Cochran’s *Q* test, MR Egger regression, and the leave-one-out method. Cochran’s *Q* test, our primary tool for identifying heterogeneity, indicates significant heterogeneity when its *p* value is below 0.05. Upon detecting significant heterogeneity, we employed a random-effects IVW model; otherwise, a fixed-effects IVW model was used. Additionally, the intercept from the MR-Egger regression provides an estimate of the extent of directional pleiotropy, with a *p* value less than 0.05 suggesting horizontal pleiotropy. The leave-one-out analysis was performed to evaluate whether the significant results were driven by a single SNP [29].

We followed the guidelines set forth by the Strengthening the Reporting of Observational Studies in Epidemiology using Mendelian Randomization (STROBE-MR) checklist [30]. *p* values reaching nominal significance (*p* < 0.05) were considered to have nominal potential causal effects. To account for multiple exposures, we adjusted the significance threshold using the Bonferroni correction, resulting in a *p*-value of < 0.0125 (= 0.05/4 exposures) considered statistically significant [31].

## Results

### Univariable MR analyses

We utilized IVs significantly correlated with T2DM and glycemic traits for two-sample MR analyses (see Supplementary Tables S2). The univariable MR analyses revealed that T2DM is positively associated with both CAC (OR_T2DM-CAC_: 1.091, 95% CI 1.030–1.157, *p* = 0.003, β0_T2DM-CAC_ = 0.088) and CAVS (OR_T2DM-CAVS_: 1.127, 95% CI 1.071–1.185, *p* = 4.130e−6, β0_T2DM-CAVS_ = 0.119). Similarly, HbA1c was found to be positively associated with CAC (OR_HbA1c-CAC_: 1.191, 95% CI 1.083–1.309, *p* = 3.033e−4, β0_HbA1c-CAC_ = 0.175) and CAVS (OR_HbA1c-CAVS_: 1.190, 95% CI 1.087–1.302, *p* = 1.620e−4, β0_HbA1c-CAVS_ = 0.174). Additionally, FG was associated with an increased risk of CAVS (OR_FG-CAVS_: 1.356, 95% CI 1.045–1.760, *p* = 0.022 > 0.0125, β0_FG-CAVS_ = 0.305). However, no causal relationship was observed between FI and vascular or valvular calcification. Furthermore, FG did not show an association with CAC risk (Table 1). There was no evidence of horizontal pleiotropy (*p* value for MR-Egger intercept test > 0.05) for T2DM, HbA1c, and FG with the risk of vascular and valvular calcifications (Table 1). The scatter plots and Leave-one-out analyses for DM and glycemic traits with the risk of both types of calcifications can be found in Supplementary Figures (Supplementary Figures S1–S10). Scatter plots intuitively display the results obtained from the four MR methods. The abscissa represents the causal effect of the IVs on the outcome, while the ordinate shows their effect on the exposure. The slopes of the lines indicate the causal effect estimated by each method.

**Table 1:** Assessing the causal relationships between DM, glycemic traits, and vascular and valvular calcifications

Expoure	Outcome	Methods	nSNP	β0	*p*	OR (95% CI)	Egger intercept (*p*value)	Cochrane *Q* (*p*value)	MR-PRESSO (*p*value)
Type 2 diabetes mellitus	Coronary artery calcification	IVW	113	0.088	0.003	1.091 (1.03, 1.157)	0.979	0.368	0.004
		MR-Egger	113	0.089	0.187	1.093 (0.959, 1.247)			
		Weighted median	113	0.079	0.143	1.083 (0.973, 1.204)			
		Weighted model	113	0.075	0.197	1.078 (0.962, 1.208)			
Type 2 diabetes mellitus	Calcific aortic valvular stenosis	IVW	111	0.119	4.130E−06	1.127 (1.071, 1.185)	0.081	0.022	1.115E−05
		MR-Egger	111	0.022	0.718	1.022 (0.907, 1.152)			
		Weighted median	111	0.057	0.127	1.059 (0.984, 1.14)			
		Weighted model	111	0.038	0.465	1.039 (0.939, 1.149)			
Glycated hemoglobin	Coronary artery calcification	IVW	261	0.175	3.033E−04	1.191 (1.083, 1.309)	0.180	0.411	3.640E−04
		MR-Egger	261	0.093	0.235	1.097 (0.942, 1.278)			
		Weighted median	261	0.080	0.386	1.083 (0.904, 1.299)			
		Weighted model	261	0.128	0.110	1.137 (0.972, 1.329)			
Glycated hemoglobin	Calcific aortic valvular stenosis	IVW	276	0.174	1.620E−04	1.190 (1.087, 1.302)	0.188	1.644E−09	1.984E−04
		MR-Egger	276	0.093	0.222	1.098 (0.945, 1.275)			
		Weighted median	276	0.118	0.072	1.126 (0.99, 1.28)			
		Weighted model	276	0.116	0.054	1.123 (0.999, 1.263)			
Fasting insulin	Coronary artery calcification	IVW	24	0.331	0.321	1.393 (0.724, 2.678)	0.856	0.027	0.331
		MR-Egger	24	0.538	0.651	1.712 (0.172, 17.009)			
		Weighted median	24	0.128	0.756	1.137 (0.508, 2.543)			
		Weighted model	24	− 0.410	0.682	0.664 (0.096, 4.586)			
Fasting insulin	Calcific aortic valvular stenosis	IVW	23	0.104	0.784	1.11 (0.526, 2.341)	0.157	5.490E−07	0.786
		MR-Egger	23	1.907	0.152	6.735 (0.544, 83.352)			
		Weighted median	23	0.158	0.694	1.171 (0.534, 2.566)			
		Weighted model	23	1.583	0.033	4.871 (1.24, 19.137)			
Fasting glucose	Coronary artery calcification	IVW	56	0.125	0.337	1.133 (0.878, 1.463)	0.564	0.465	0.341
		MR-Egger	56	0.005	0.984	1.005 (0.622, 1.624)			
		Weighted median	56	− 0.030	0.887	0.97 (0.64, 1.471)			
		Weighted model	56	0.021	0.926	1.022 (0.654, 1.594)			
Fasting glucose	Calcific aortic valvular stenosis	IVW	54	0.305	0.022	1.356 (1.045, 1.760)	0.897	1.314E−04	0.026
		MR-Egger	54	0.331	0.174	1.392 (0.87, 2.229)			
		Weighted median	54	0.339	0.039	1.404 (1.016, 1.939)			
		Weighted model	54	0.362	0.014	1.437 (1.088, 1.898)			

### Reverse MR analyses

Next, we conducted a reverse MR analysis. Supplementary Tables S3 showed a causal effect of CAVS on FG (OR_CAVS-FG_: 1.010, 95% CI 1.002–1.018, *p* = 0.015). However, no causal relationships were observed between vascular or valvular calcification and T2DM, HbA1c, or FI. Similarly, no significant causal effect of CAC on FG was detected (see Supplementary Tables S3 and S4 for further details). There was no evidence of horizontal pleiotropy (*p* value for the MR-Egger intercept test > 0.05) for CAVS on FG (Table S3). The scatter plots and Leave-one-out analyses for CAVS on FG can be found in Supplementary Figures S11 and S12.

### Two-step MR and mediation analysis

We subsequently explored the association between T2DM, HbA1c, and FG with calcium homeostasis, including serum calcium, 25OHD, and FGF23, to identify potential intermediaries in the pathway linking DM and glycemic traits to vascular and valvular calcifications, which have previously demonstrated a causal relationship. The IVW MR analysis revealed significant associations: (1) T2DM was associated with increased calcium levels (OR_T2DM-Ca_: 1.034, 95% CI 1.021–1.047, *p* = 3.62e−7, β1_T2DM-Ca_ = 0.033) and decreased 25OHD (OR_T2DM-25OHD_: 0.968, 95% CI 0.959–0.978, *p* = 2.343e−10, β1_T2DM-25OHD_ = − 0.032); (2) HbA1c was linked with an increase in calcium (OR_HbA1c-Ca_: 1.059, 95% CI 1.029–1.091, *p* = 1.223e−04, β1_HbA1c-Ca_ = 0.057); 3) FG showed a significant rise in calcium (OR_FG-Ca_: 1.222, 95% CI 1.099–1.359, *p* = 2.165e−04, β1_FG-Ca_ = 0.200) and a decrease in 25OHD (OR_FG-25OHD_: 0.897, 95% CI 0.863–0.934, *p* = 8.383e−08, β1_FG-25OHD_ = − 0.108). Results for other tests were negative and are not presented here; see Supplementary Tables S5 and S6 for further details. No evidence of horizontal pleiotropy was found (*p* value for the MR-Egger intercept test > 0.05) for T2DM, HbA1c, and FG on calcium homeostasis (Table S5). The scatter plots and Leave-one-out analyses for T2DM, HbA1c, and FG on calcium homeostasis are presented in Supplementary Figures S13–S22.

We then investigated the association of calcium homeostasis with CAC and CAVS. Of the components studied, only calcium levels were associated with both CAC (OR_Ca-CAC_: 1.173, 95% CI 1.016–1.354, *p* = 0.030, β2_Ca-CAC_ = 0.159) and CAVS (OR_Ca-CAVS_: 1.151, 95% CI 1.021–1.298, *p* = 0.021, β2_Ca-CAVS_ = 0.141). The remaining negative results are not presented, see Supplementary Tables S7 and S8 for details. Similarly, no evidence of horizontal pleiotropy was found for calcium levels on both types of calcifications (Table S7). The scatter plots and Leave-one-out analyses for calcium levels on both types of calcifications are presented in Supplementary Figures S23–S26.

**Fig. 2:**
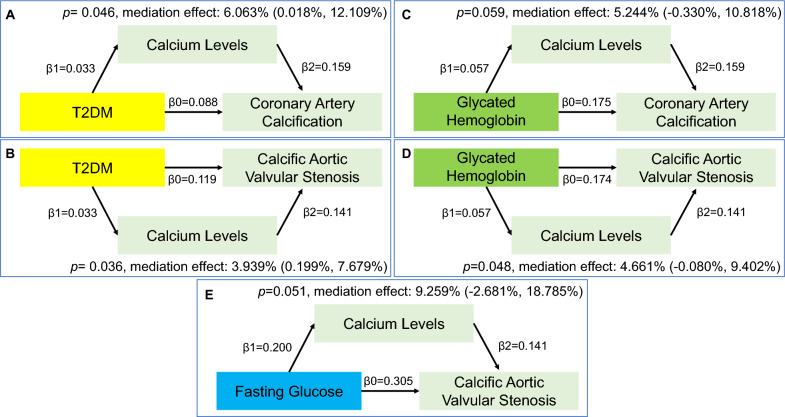
The mediation effect of calcium levels in the relationships between DM, glycemic traits, and vascular and valvular calcifications. Calcium levels mediated the impact of T2DM on CAC and CAVS were 6.063% (**A**) and 3.939% (**B**), respectively. No mediating effects of calcium levels were observed between HbA1c and FG on both types of calcifications (**C**–**E**)

The mediation analysis uncovered that calcium levels play a partial mediating role in the causal pathways from T2DM to CAC, with a mediation effect of 6.063% (95% CI 0.018–12.109%, *p* = 0.046), and from T2DM to CAVS, with a mediation effect of 3.939% (95% CI 0.199–7.679%, *p* = 0.036). However, HbA1c's influence on CAC showed a mediation effect of 5.244% (95% CI − 0.330–10.818%, *p* = 0.059), and on CAVS, a mediation effect of 4.661% (95% CI − 0.080–9.402%, *p* = 0.048). For FG's impact on CAVS, the mediation effect was 9.259% (95% CI − 2.681–18.785%, *p* = 0.051). See Fig. 2 for details.

It's crucial to note that only mediator traits showing a causal effect in the initial analysis and passing the sensitivity analysis are eligible for the second step of the two-step MR process. Figure 2A–E illustrates the findings from the two-step MR analysis, employing IVW as the principal analytical method, along with the results of the mediation analysis.

### Multivariable MR

Finally, we employed multivariable MR to evaluate the associations between T2DM, HbA1c and FG with both vascular and valvular calcifications, while accounting for potential cardiometabolic risk factors such as BMI, smoking initiation, systolic blood pressure, and diastolic blood pressure.

The relationship between T2DM and CAC remained significant when controlling for BMI (OR: 1.088, 95% CI 1.016–1.164, *p* = 0.015) or smoking initiation (OR: 1.112, 95% CI 1.031–1.200, *p* = 0.006). However, after adjusting for systolic blood pressure (OR: 1.087, 95% CI 0.931–1.269, *p* = 0.290) or diastolic blood pressure (OR: 1.100, 95% CI 0.963–1.256, *p* = 0.159), the association with CAC risk was no longer significant.

For CAVS, the effect of T2DM remained robust when controlling for smoking initiation (OR: 1.127, 95% CI 1.068–1.190, *p* = 1.428e−5). However, the significance was lost when adjusting for BMI (OR: 1.062, 95% CI 1.000–1.127, *p* = 0.050), systolic blood pressure (OR: 1.077, 95% CI 0.980–1.183, *p* = 0.123), or diastolic blood pressure (OR: 1.067, 95% CI 0.970–1.174, *p* = 0.183).

The association between HbA1c and CAC was strong when controlling for BMI (OR: 1.203, 95% CI 1.070–1.352, *p* = 0.002) or smoking initiation (OR: 1.208, 95% CI 1.075–1.358, *p* = 0.002). However, adjustments for systolic blood pressure (OR: 1.225, 95% CI 0.804–1.865, *p* = 0.345) or diastolic blood pressure (OR: 1.232, 95% CI 0.871–1.743, *p* = 0.239) rendered the relationship with CAC risk insignificant.

Regarding CAVS, the effect of HbA1c was substantial when accounting for BMI (OR: 1.166, 95% CI 1.054–1.291, *p* = 0.003), smoking initiation (OR: 1.196, 95% CI 1.077–1.327, *p* = 0.001), or diastolic blood pressure (OR: 1.303, 95% CI 1.013–1.676, *p* = 0.039), However, significance was lost after adjusting for systolic blood pressure (OR: 1.214, 95% CI 0.939–1.570, *p* = 0.139).

The influence of FG on CAVS was significant when controlling for BMI (OR: 1.355, 95% CI 1.070–1.717, *p* = 0.012) or smoking initiation (OR: 1.391, 95% CI 1.069–1.810, *p* = 0.014). However, the association became non-significant after adjustments for systolic blood pressure (OR: 1.304, 95% CI: 0.847–2.007, *p* = 0.227) or diastolic blood pressure (OR: 1.351, 95% CI: 0.889–2.053, *p* = 0.158). See Table 2 for details. Furthermore, no evidence of horizontal pleiotropy was found in the multivariable MR results after adjusting for cardiometabolic risk factors, as indicated by *p* value greater than 0.05 in the MR-Egger intercept test (Table 2).

**Table 2:** Multivariable MR results after adjusting for cardiometabolic risk factors

Exposure	Outcome	Adjustment	nSNP	Methods	Causal effect		Heterogeneity		Pleiotropy	
					*p*	OR (95% CI)	*Q*	*p* value	Intercept	*p* value
Type 2 Diabetes Mellitus	Coronary artery calcification	Body mass index	364	IVW	0.015	1.088 (1.016, 1.164)	413.444	0.032	− 3.492E−04	0.879
		Smoking initiation	151	IVW	0.006	1.112 (1.031, 1.200)	231.520	1.719E−05	0.001	0.892
		Systolic blood pressure	157	IVW	0.290	1.087 (0.931, 1.269)	503.957	1.620E−38	− 0.003	0.441
		Diastolic blood pressure	160	IVW	0.159	1.100 (0.963, 1.256)	378.966	3.410E−20	− 0.001	0.795
Type 2 Diabetes Mellitus	Calcific aortic valvular stenosis	Body mass index	353	IVW	0.050	1.062 (1.000, 1.127)	523.674	5.896E−09	− 0.002	0.249
		Smoking initiation	148	IVW	1.428E−05	1.127 (1.068, 1.190)	195.103	0.004	0.002	0.538
		Systolic blood pressure	156	IVW	0.123	1.077 (0.980, 1.183)	305.133	5.044E−12	0.001	0.757
		Diastolic blood pressure	159	IVW	0.183	1.067 (0.970, 1.174)	320.335	2.951E−13	− 2.339E−03	0.317
Glycated Hemoglobin	Coronary artery calcification	Body mass index	455	IVW	0.002	1.203 (1.070, 1.352)	600.725	3.750E−06	0.001	0.495
		Smoking initiation	244	IVW	0.002	1.208 (1.075, 1.358)	319.206	0.001	3.238E−04	0.901
		Systolic blood pressure	123	IVW	0.345	1.225 (0.804, 1.865)	353.991	1.075E−24	0.002	0.766
		Diastolic blood pressure	110	IVW	0.239	1.232 (0.871, 1.743)	211.651	1.016E−08	− 3.471E−04	0.956
Glycated hemoglobin	Calcific aortic valvular stenosis	Body mass index	447	IVW	0.003	1.166 (1.054, 1.291)	725.518	8.713E−16	0.002	0.171
		Smoking initiation	245	IVW	0.001	1.196 (1.077, 1.327)	417.938	2.072E−11	1.108E−04	0.961
		Systolic blood pressure	124	IVW	0.139	1.214 (0.939, 1.570)	269.202	4.151E−13	− 0.008	0.087
		Diastolic blood pressure	112	IVW	0.039	1.303 (1.013, 1.676)	228.963	2.295E−10	− 0.009	0.055
Fasting glucose	Calcific aortic valvular stenosis	Body mass index	414	IVW	0.012	1.355 (1.070, 1.717)	655.700	2.072E−13	0.001	0.509
		Smoking initiation	126	IVW	0.014	1.391 (1.069, 1.810)	241.739	1.322E−09	0.005	0.060
		Systolic blood pressure	191	IVW	0.227	1.304 (0.847, 2.007)	413.954	7.218E−19	− 0.003	0.474
		Diastolic blood pressure	180	IVW	0.158	1.351 (0.889, 2.053)	362.893	9.646E−15	− 0.003	0.487

## Discussion

Our findings reveal complex interactions between DM, glycemic traits, and vascular and valvular calcifications, and highlight the mediating role of calcium homeostasis in these associations. The results demonstrate that T2DM and HbA1c are positively associated with both CAC and CAVS. The associations between T2DM and both CAC and CAVS are mediated by disturbances in calcium levels. Furthermore, the associations between T2DM and HbA1c with both types of calcifications, as well as FG with CAVS, were modified by BMI (except for T2DM with CAVS, *p* = 0.050) and smoking status. However, these associations were generally reduced after adjusting for systolic blood pressure and diastolic blood pressure (except for HbA1c with CAVS, *p* = 0.039). This study underscores a crucial link between metabolic dysregulation and the progression of cardiovascular calcifications.

The observed associations between DM and cardiovascular calcifications align with the growing body of literature suggesting that metabolic syndromes contribute to cardiovascular risks [9, 11, 32]. On the other hand, our study aligns with existing literature that identifies HbA1c levels as predictors of cardiovascular calcifications. For instance, the progression of CAC has been associated with higher HbA1c levels even in individuals without diabetes, highlighting the broad relevance of glycemic control in cardiovascular health [33]. In addition, a cross-sectional study with a large population showed that HbA1c is associated with incidences of elevated CAC scores measured by computed tomography (CT) scan [3]. This suggests that glycemic control may be crucial not only for diabetes management but also for the prevention of cardiovascular calcifications. Furthermore, research indicates that variability in HbA1c levels, rather than just elevated averages, might also predict cardiovascular outcomes in diabetes. Higher HbA1c levels were associated with greater incident CAC and CAVS progression after adjusting for sociodemographic factors. The association between HbA1C and CAC progression persisted in multivariable-adjusted models [34]. Interestingly, Cardoso et al. found that long-term visit-to-visit FG variability was a better predictor than mean HbA1c for assessing the risk of future development of micro- and macrovascular complications [35]. These associations suggest that glycemic instability itself could contribute to vascular pathologies, although we only found that FG was associated with an increased risk of CAVS (OR: 1.356, 95% CI 1.045–1.760, *p* = 0.022 > 0.0125).

Calcium homeostasis plays a crucial role in various metabolic and cardiovascular processes. Recent studies highlight its involvement in the pathogenesis of cardiovascular diseases by mitochondria, particularly in the context of DM and its associated glycemic traits [36]. On the other hand, DM and its related glycemic conditions are well-documented risk factors for cardiovascular diseases, including CAC and CAVS [5]. Our analyses reveal the significance of serum calcium in mediating the relationship between T2DM and vascular and valvular calcifications. By quantifying the mediation effect of altered calcium levels, we provide a potential pathway through which glycemic control may influence CAC and CAVS. This aligns with research showing that disrupted calcium metabolism in diabetes and chronic kidney disease patients contributes to vascular calcification [37]. Notably, decreased serum levels of 25OHD have been linked to increased vascular calcification risks, further substantiated by findings associating low vitamin D levels with poor cardiovascular outcomes [37]. However, in our study, calcium levels partially mediated the impact of T2DM on both types of calcifications, while 25OHD did not exhibit a mediation effect. Moreover, FGF23, a hormone known for its roles in phosphate and vitamin D metabolism, has been implicated in the pathogenesis of cardiovascular calcifications, particularly through its association with vascular calcification. Wungu et al. indicated that FGF23 was associated with arterial calcification, thickness, and stiffness, clarifying their role in arterial remodeling processes [38]. Our findings do not establish a direct causal relationship between FGF23 levels and cardiovascular calcifications in the general population. This discrepancy underscores the complexity of metabolic interactions in cardiovascular calcifications and highlights the potential for differential impacts across patient populations with varying degrees of renal function and metabolic health [37]. A quantitative measure is provided by the mediation effects observed for calcium on the relationship between T2DM and both types of calcifications. This measure shows the extent to which calcium homeostasis impacts this relationship. Significant clinical implications arise from this finding. It suggests that managing calcium levels could mitigate some of the cardiovascular risks associated with diabetes.

For modifiable cardiometabolic risk factors, Jensen et al. reported that in a large sample undergoing CAC scoring, obesity was associated with a higher risk of CAC and subsequent coronary heart disease, CVD, and all-cause mortality [39]. Moreover, Shaw et al. demonstrated that young smokers with high-risk coronary artery calcium scores have a four- to nine-fold increased risk of dying when compared with similarly aged non-smokers [40]. In this study, the associations between T2DM and HbA1c with both types of calcifications, and FG with CAVS, were modified by BMI (except for T2DM with CAVS, *p* = 0.050) and smoking initiation. Additionally, Kramer et al. (2009) concluded that in older adults without known heart disease, blood pressure levels were better independent determinants of CAC progression than metabolic syndrome itself [41]. The finding is consistent with our study, which showed that associations between T2DM and HbA1c with both types of calcifications generally reduced after adjusting for blood pressure (except for HbA1c with CAVS adjusting for diastolic blood pressure, *p* = 0.039). This suggests that while glycemic control is crucial, overall cardiovascular risk management remains complex and multifactorial.

Limitations should be considered. First, although MR offers a robust method to estimate causal effects by using genetic variants as IVs, the validity of the results depends crucially on these assumptions that the IVs affect the outcome solely through the exposure (T2DM and glycemic traits) and are not associated with any confounders. Any pleiotropy or linkage disequilibrium with other loci affecting the outcome could bias the estimates. Second, the study's findings are based on genetic data that may not be entirely representative of all populations. The genetic variants used as IVs were primarily identified in populations of European ancestry. This raises concerns about the generalizability of the findings to other ethnic groups, who may have different prevalence rates of DM, glycemic traits, and cardiovascular calcifications, and possibly different genetic architectures. Third, calcium homeostasis was assessed through serum calcium, 25OHD, and FGF23. However, these measurements can be influenced by various factors not accounted for in this study, such as dietary intake, parathyroid function, and renal function, which may lead to misclassification and bias. Fourth, despite adjustments for numerous confounders via multivariable MR, the possibility of unmeasured confounding persists. Lifestyle behaviors, socioeconomic status, and other environmental factors might influence both calcium homeostasis and cardiovascular calcifications but were not comprehensively captured in the genetic analysis.

## Conclusion

In conclusion, this study not only confirms the significant associations between DM, glycemic traits, and vascular and valvular calcifications but also highlights the crucial role of serum calcium in mediating these effects. The insights garnered from our comprehensive MR approach could guide future clinical strategies aimed at the simultaneous management of metabolic and cardiovascular calcifications, potentially improving outcomes for patients with DM. Further research, especially longitudinal studies and clinical trials, is essential to validate these findings and explore the underlying mechanisms in more detail.

### Supplementary Information

Supplementary Material 1.

Supplementary Material 2.

Supplementary Material 3.

Supplementary Material 4.

Supplementary Material 5.

Supplementary Material 6.

Supplementary Material 7.

Supplementary Material 8.

Supplementary Material 9.

Supplementary Material 10.

Supplementary Material 11.

Supplementary Material 12.

Supplementary Material 13.

Supplementary Material 14.

Supplementary Material 15.

Supplementary Material 16.

Supplementary Material 17.

Supplementary Material 18.

Supplementary Material 19.

Supplementary Material 20.

Supplementary Material 21.

Supplementary Material 22.

Supplementary Material 23.

Supplementary Material 24.

Supplementary Material 25.

Supplementary Material 26.

Supplementary Material 27.

Supplementary Material 28.

## Data Availability

No datasets were generated or analysed during the current study.

## Abbreviations

25OHD: 25-Hydroxyvitamin D
BMI: Body mass index
CAC: Coronary artery calcification
CAVS: Calcific aortic valvular stenosis
CI: Confidence interval
CT: Computed tomography
CVD: Cardiovascular disease
DM: Diabetes mellitus
FG: Fasting glucose
FGF23: Fibroblast growth factor 23
FI: Fasting insulin
GWAS: Genome-Wide Association Studies
HbA1c: Glycated hemoglobin
IVs: Instrumental variables
IVW: Inverse variance weighted
LASSO: Least absolute shrinkage and selection operator
MACE: Major cardiac adverse events
MR: Mendelian randomization
OR: Odds ratio
SNPs: Single nucleotide polymorphisms
T2DM: Type 2 diabetes mellitus
