# Supplementary figures and images for: Exploring the mediating role of calcium homeostasis in the association between diabetes mellitus, glycemic traits, and vascular and valvular calcifications: a comprehensive Mendelian randomization analysis
Source: Diabetol Metab Syndr. 2024 Jun 22;16:136. doi: 10.1186/s13098-024-01383-z (PMC11193216; doi:10.1186/s13098-024-01383-z)

### MR Test

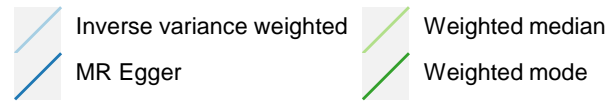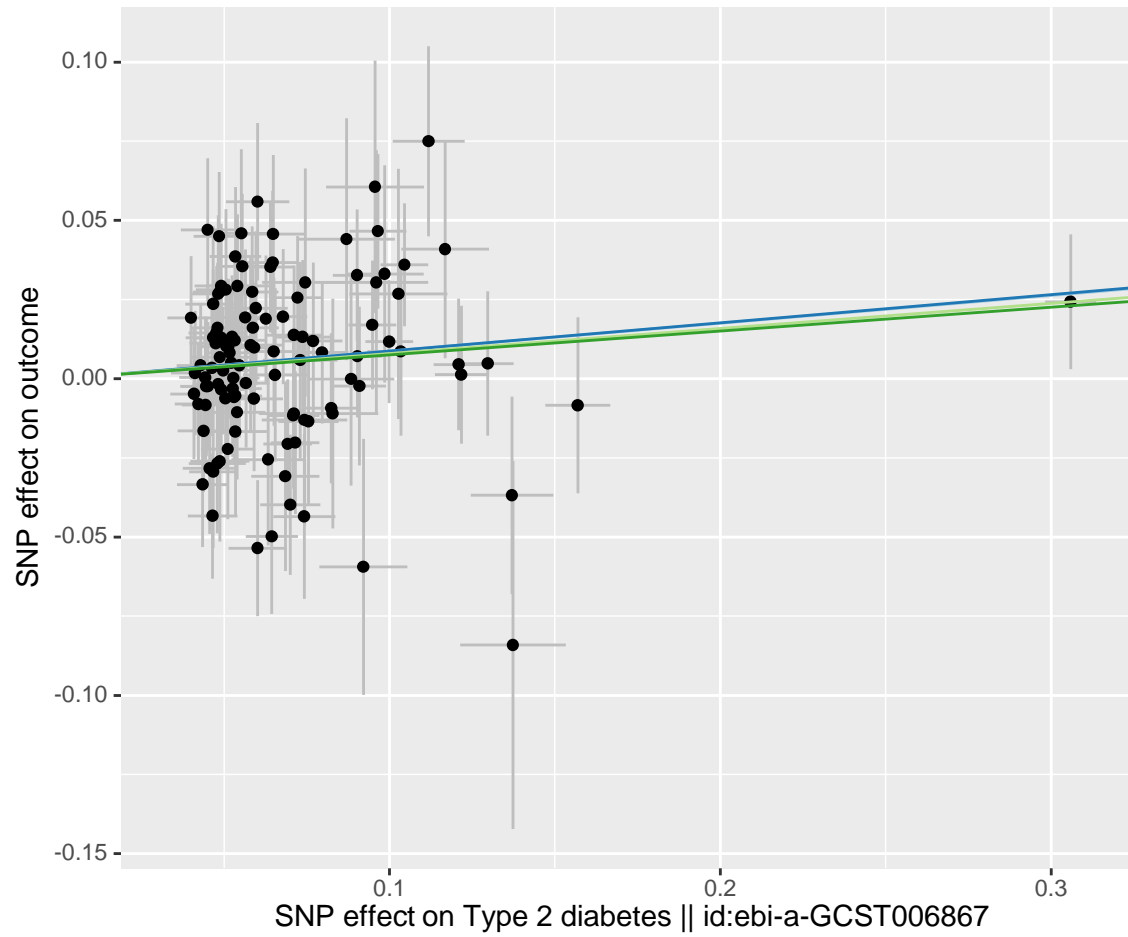

Supplement: Supplementary file 1 — Supplementary Material 1. [file 13098_2024_1383_MOESM1_ESM.pdf]

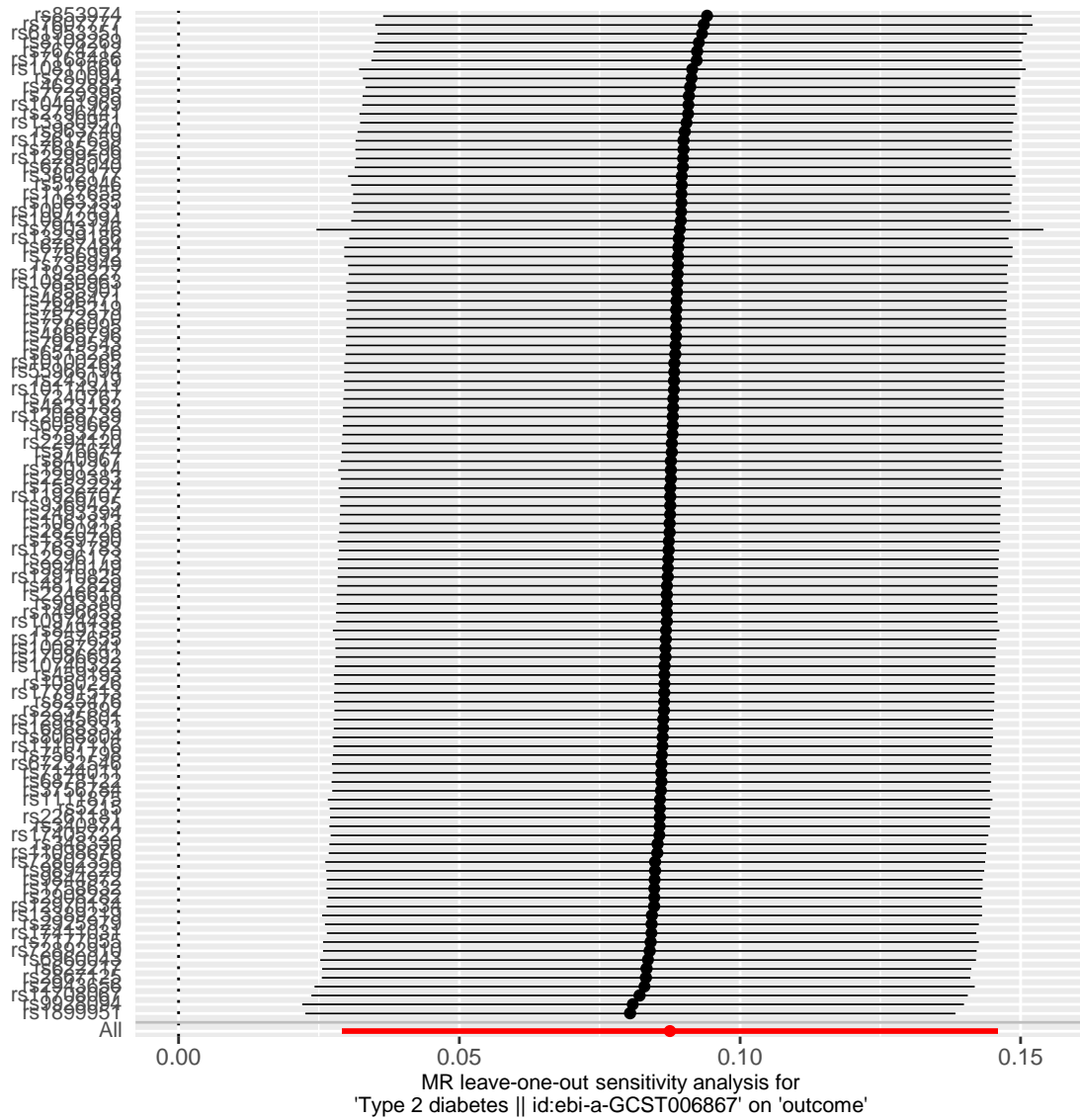

Supplement: Supplementary file 2 — Supplementary Material 2. [file 13098_2024_1383_MOESM2_ESM.pdf]

# MR Test

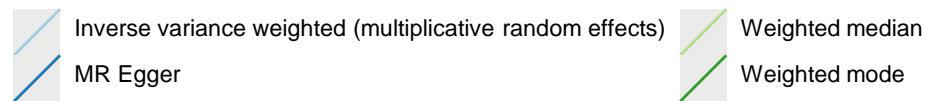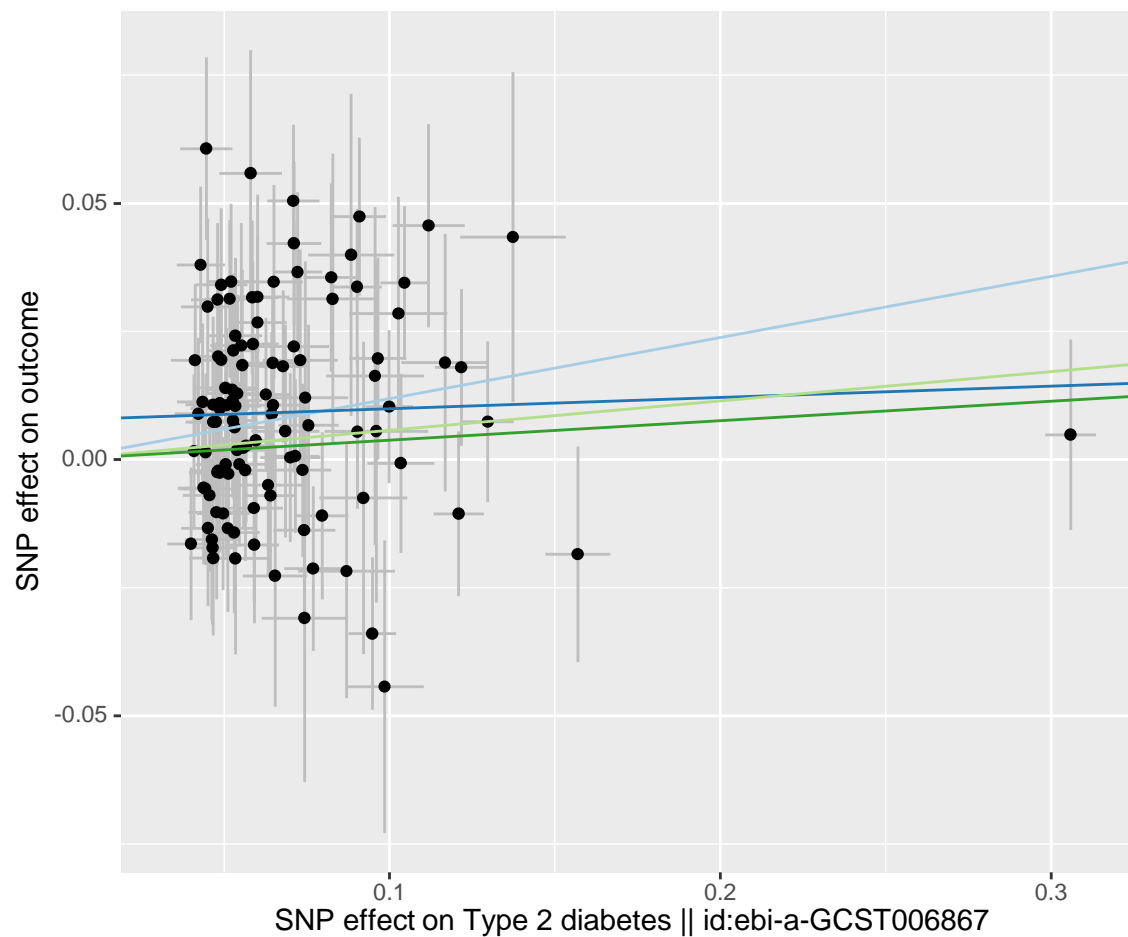

Supplement: Supplementary file 3 — Supplementary Material 3. [file 13098_2024_1383_MOESM3_ESM.pdf]

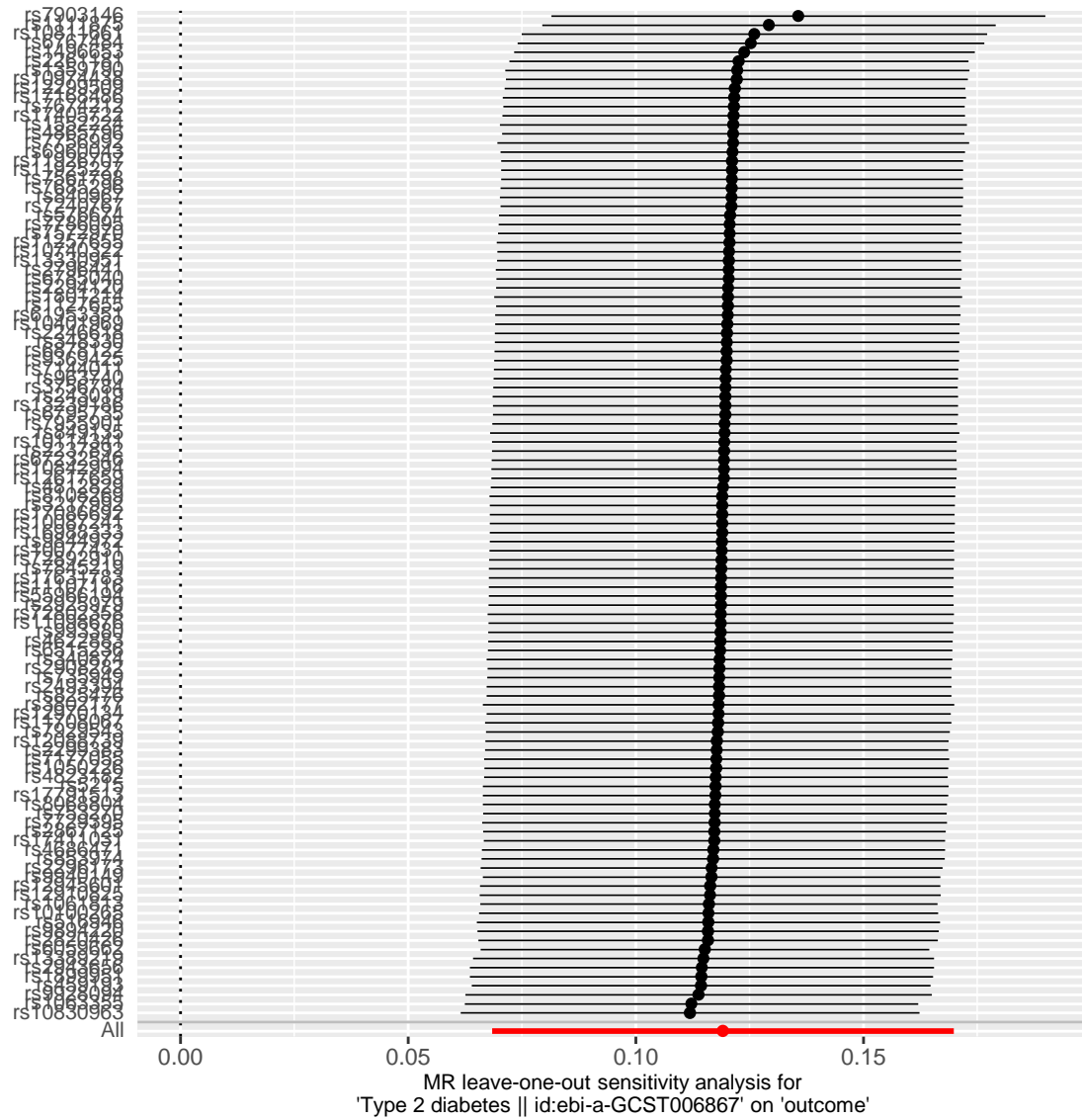

Supplement: Supplementary file 4 — Supplementary Material 4. [file 13098_2024_1383_MOESM4_ESM.pdf]

# MR Test

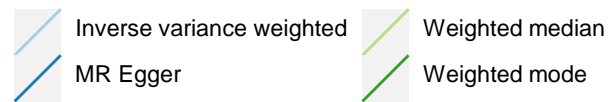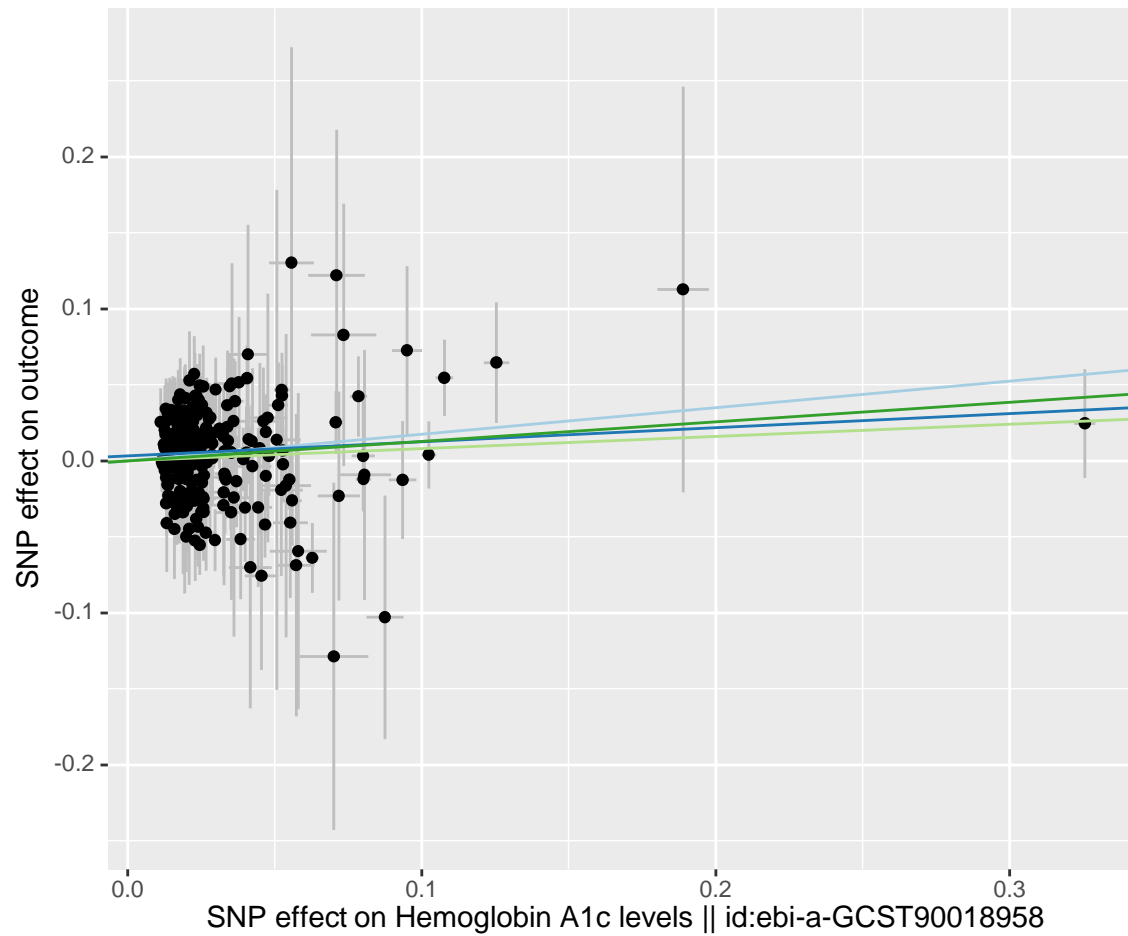

Supplement: Supplementary file 5 — Supplementary Material 5. [file 13098_2024_1383_MOESM5_ESM.pdf]

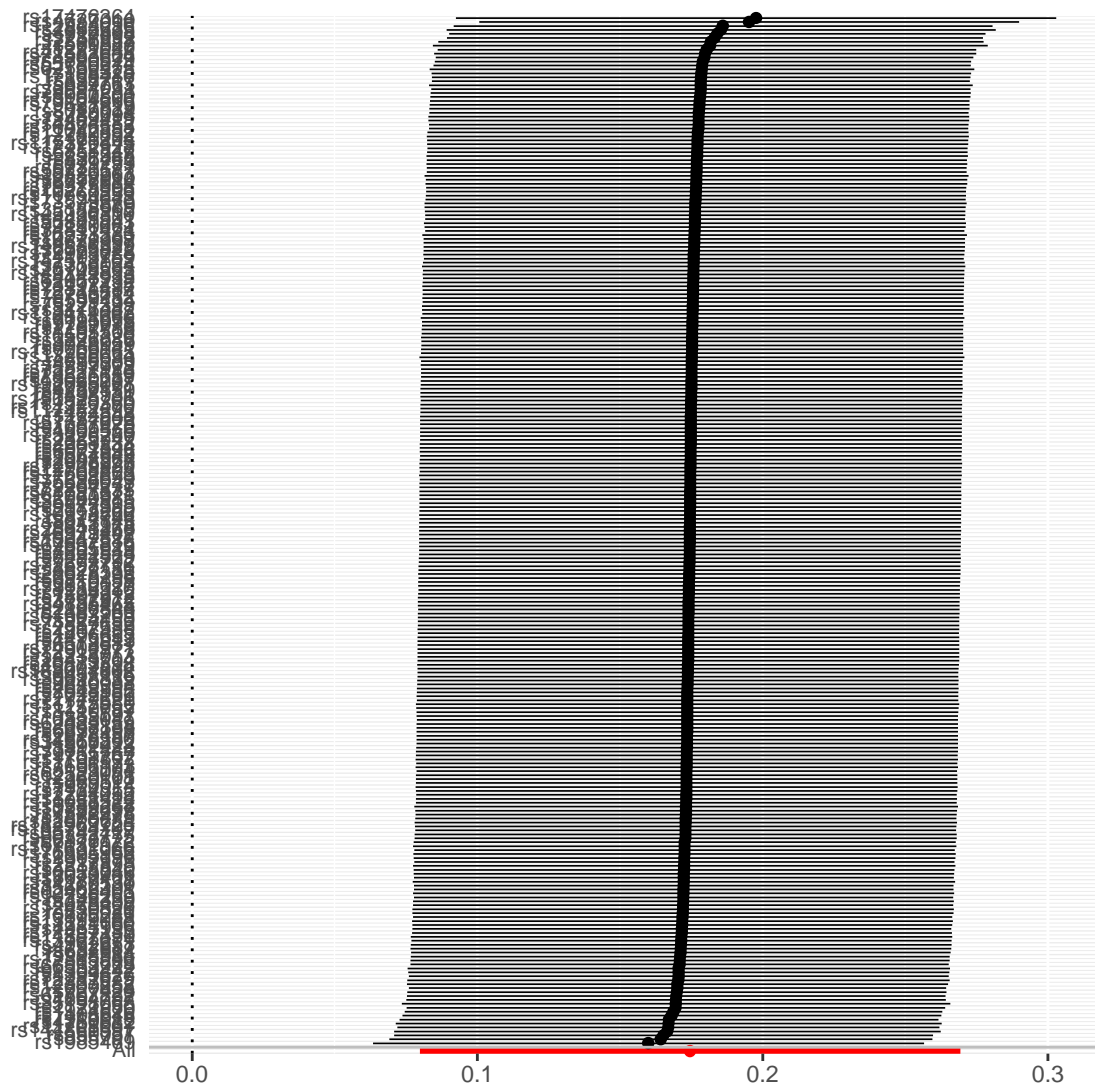

Supplement: Supplementary file 6 — Supplementary Material 6. [file 13098_2024_1383_MOESM6_ESM.pdf]

# MR Test

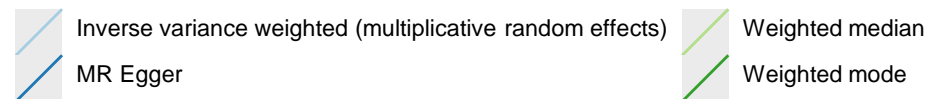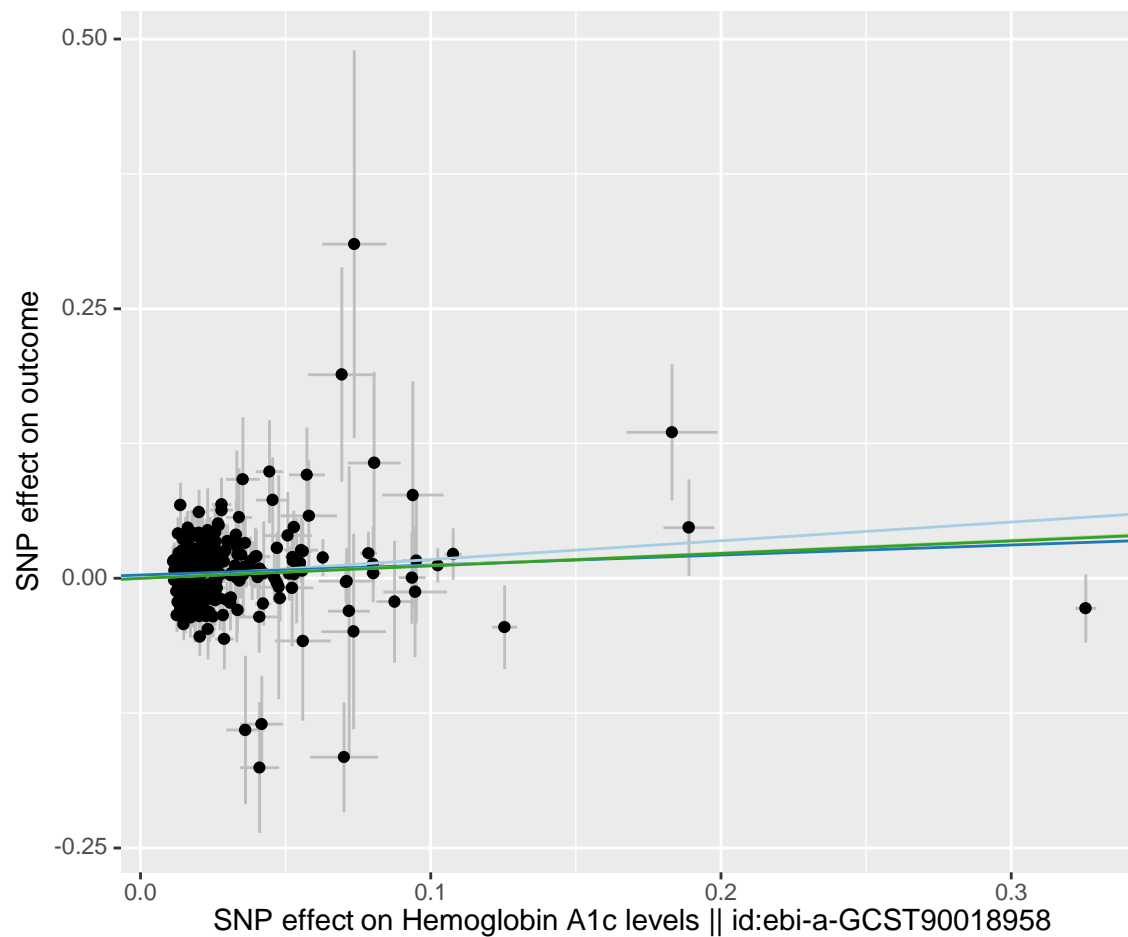

Supplement: Supplementary file 7 — Supplementary Material 7. [file 13098_2024_1383_MOESM7_ESM.pdf]

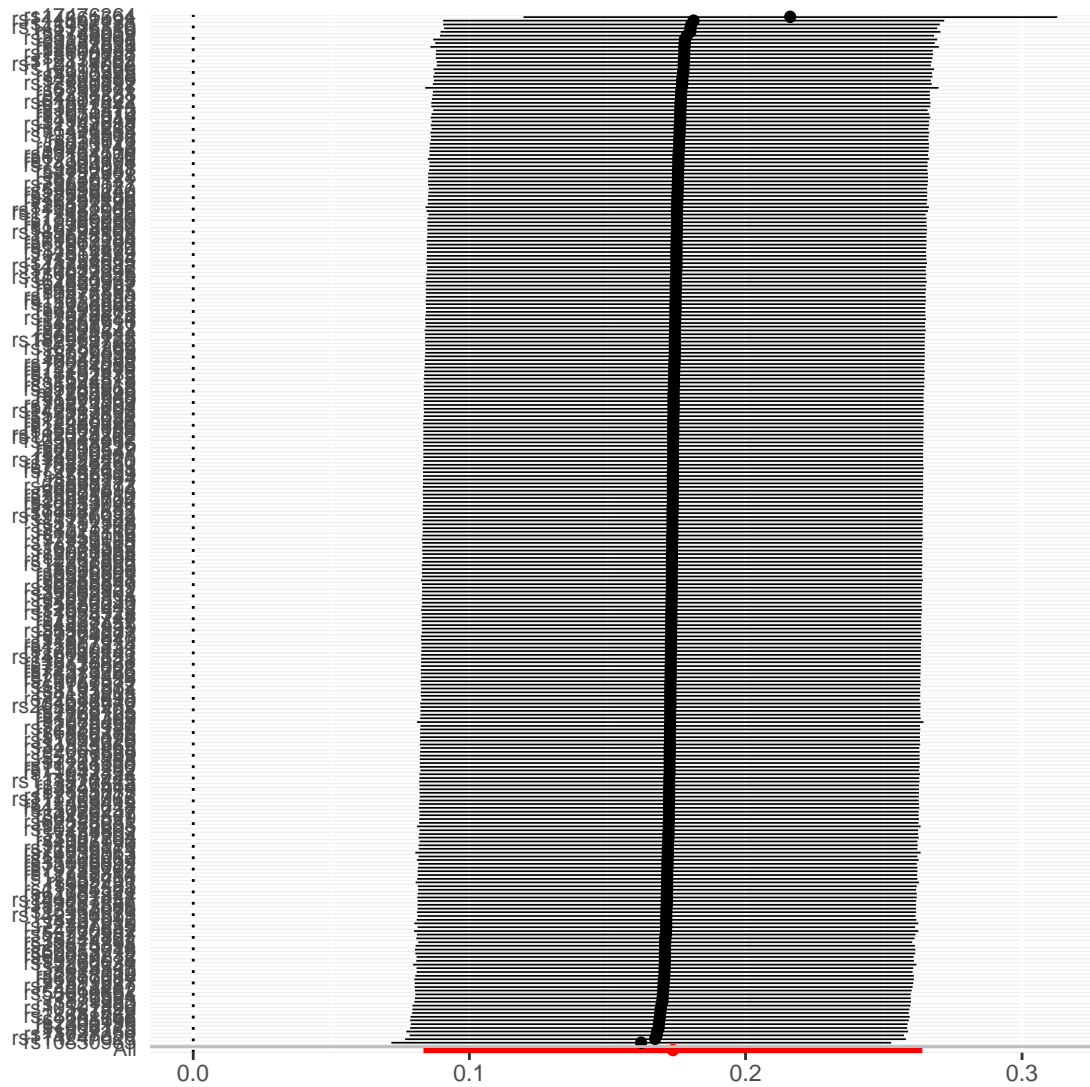

MR leave-one-out sensitivity analysis for  
'Hemoglobin A1c levels || id:ebi-a-GCST90018958' on 'outcome'

Supplement: Supplementary file 8 — Supplementary Material 8. [file 13098_2024_1383_MOESM8_ESM.pdf]

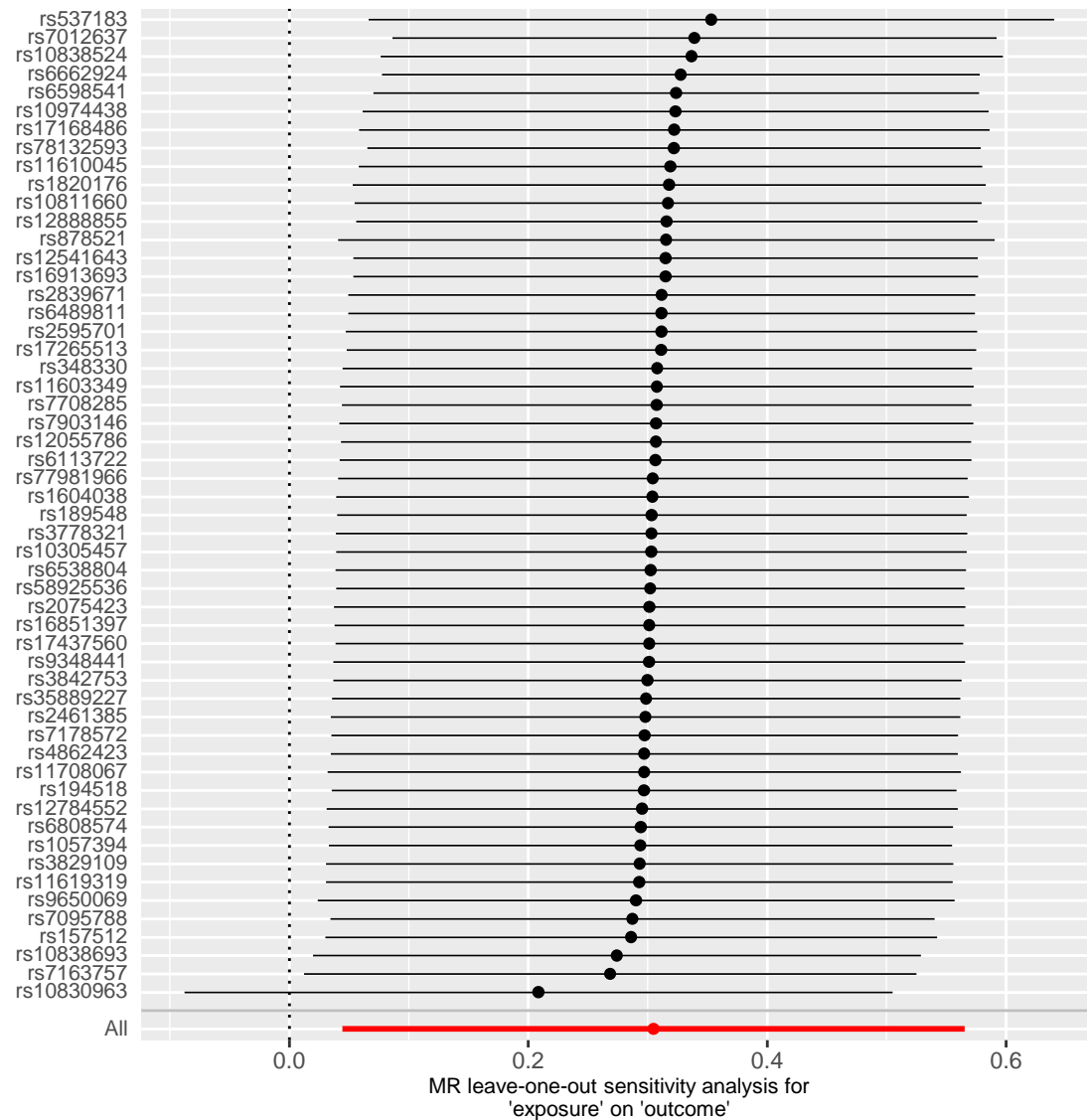

Supplement: Supplementary file 10 — Supplementary Material 10. [file 13098_2024_1383_MOESM10_ESM.pdf]

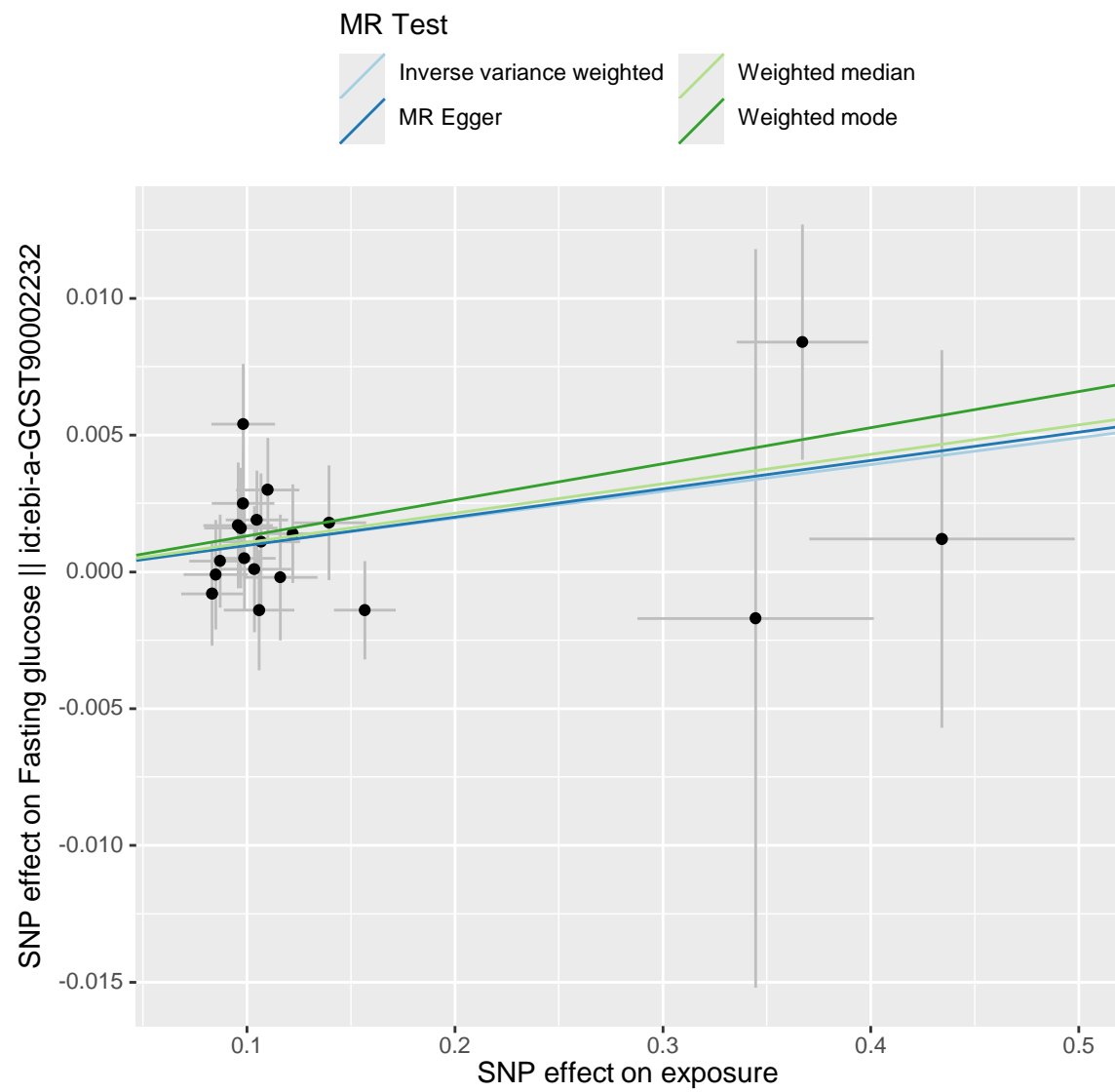

Supplement: Supplementary file 11 — Supplementary Material 11. [file 13098_2024_1383_MOESM11_ESM.pdf]

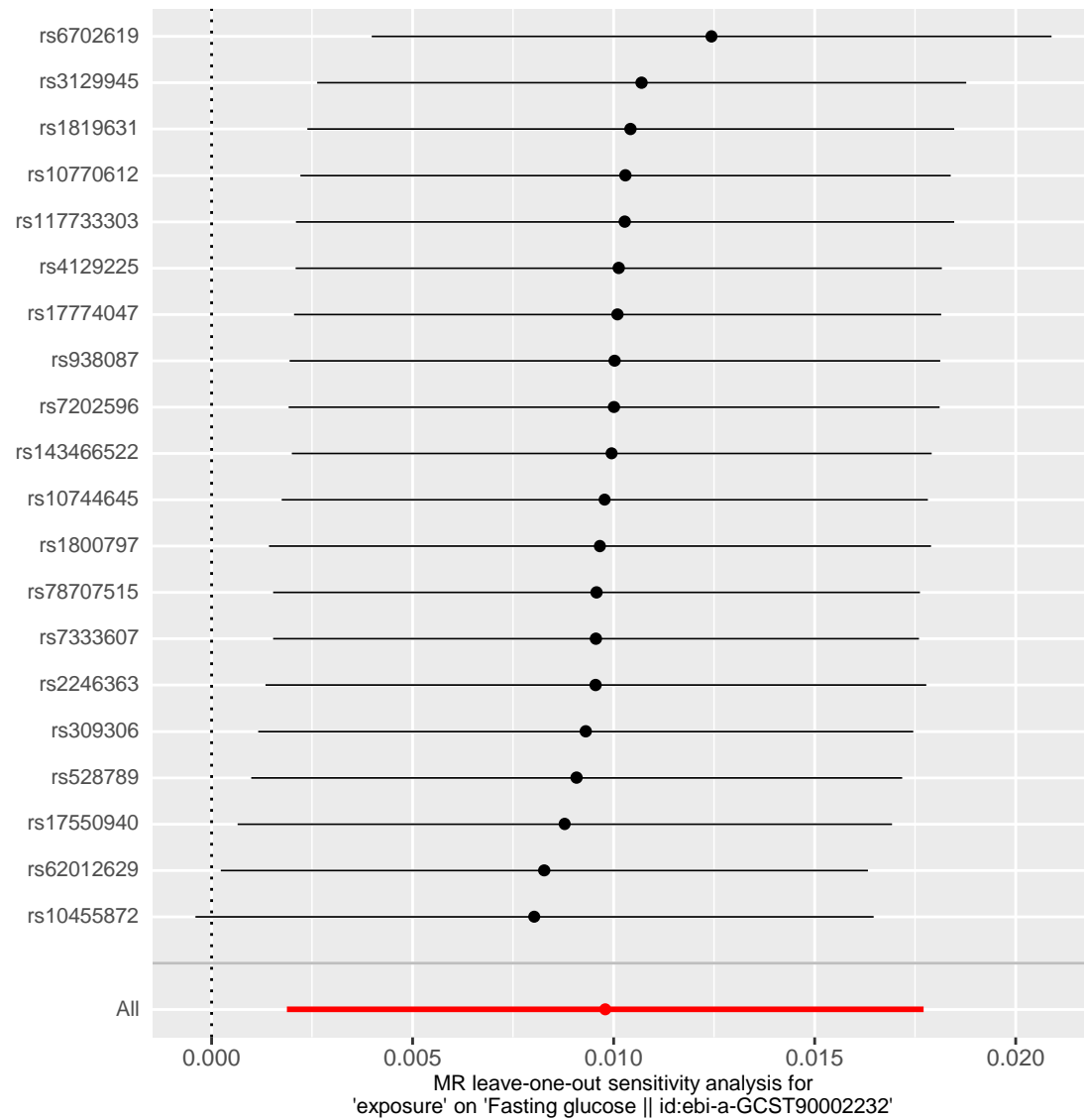

Supplement: Supplementary file 12 — Supplementary Material 12. [file 13098_2024_1383_MOESM12_ESM.pdf]

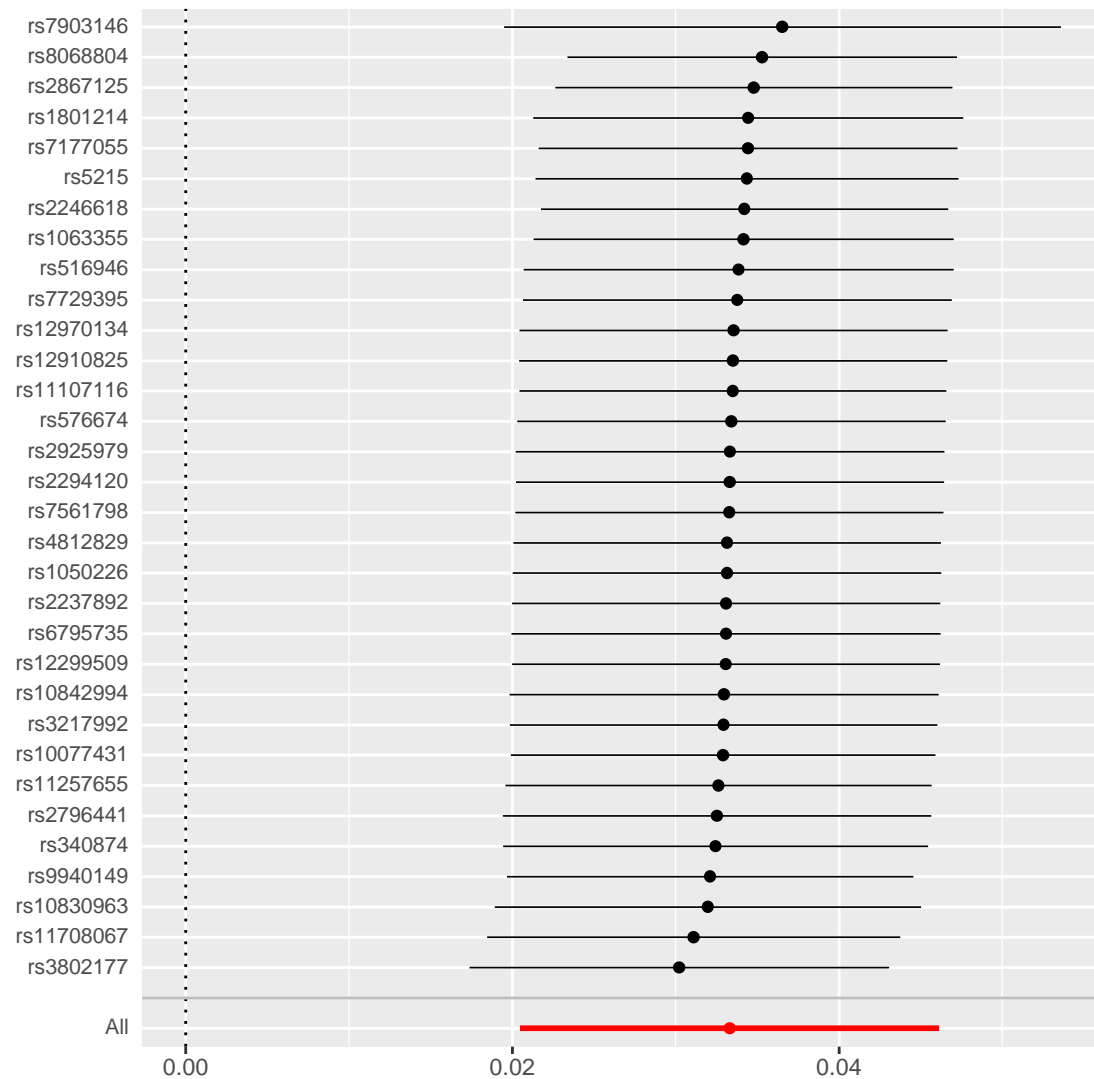

Supplement: Supplementary file 14 — Supplementary Material 14. [file 13098_2024_1383_MOESM14_ESM.pdf]

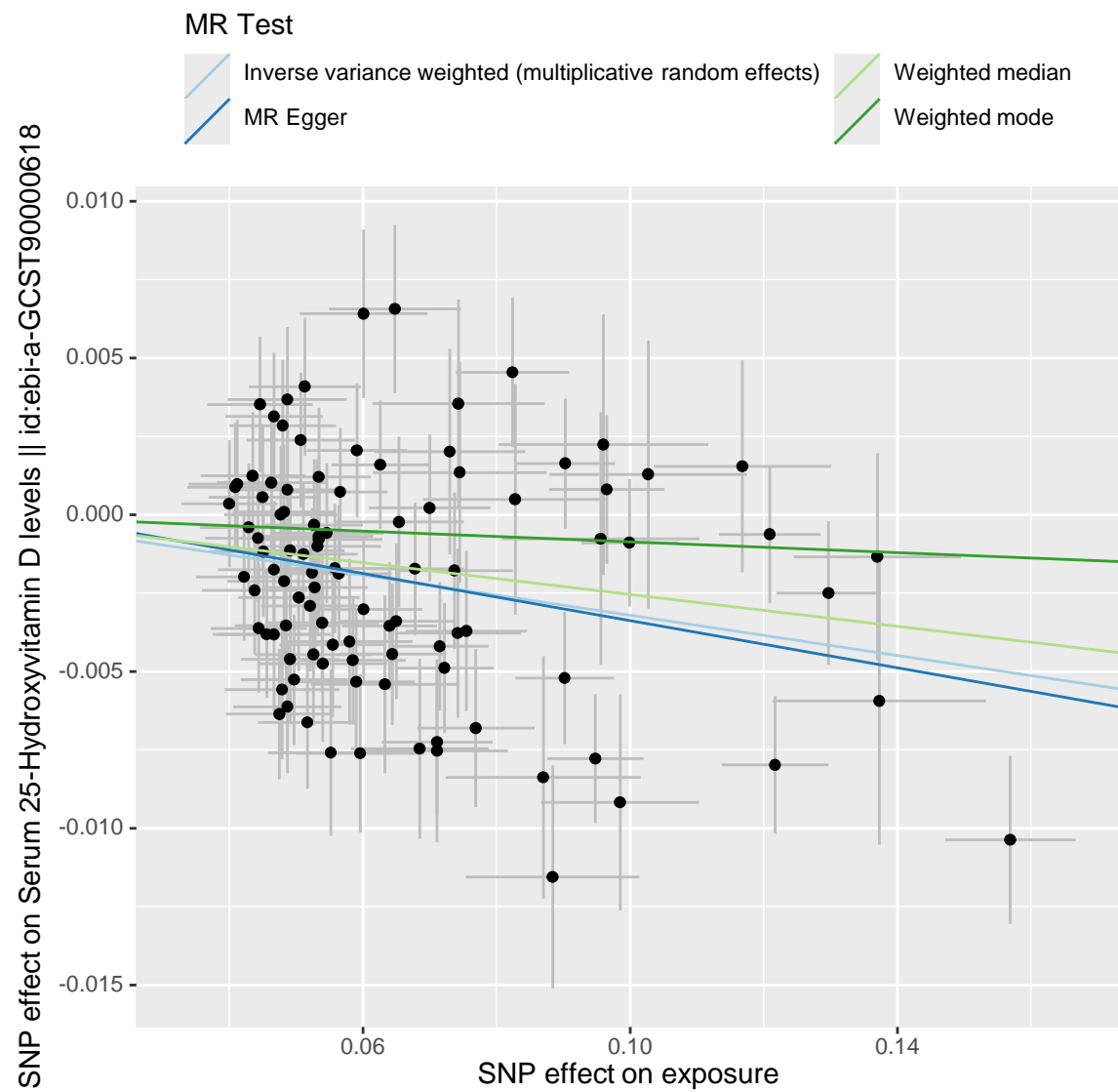

Supplement: Supplementary file 15 — Supplementary Material 15. [file 13098_2024_1383_MOESM15_ESM.pdf]

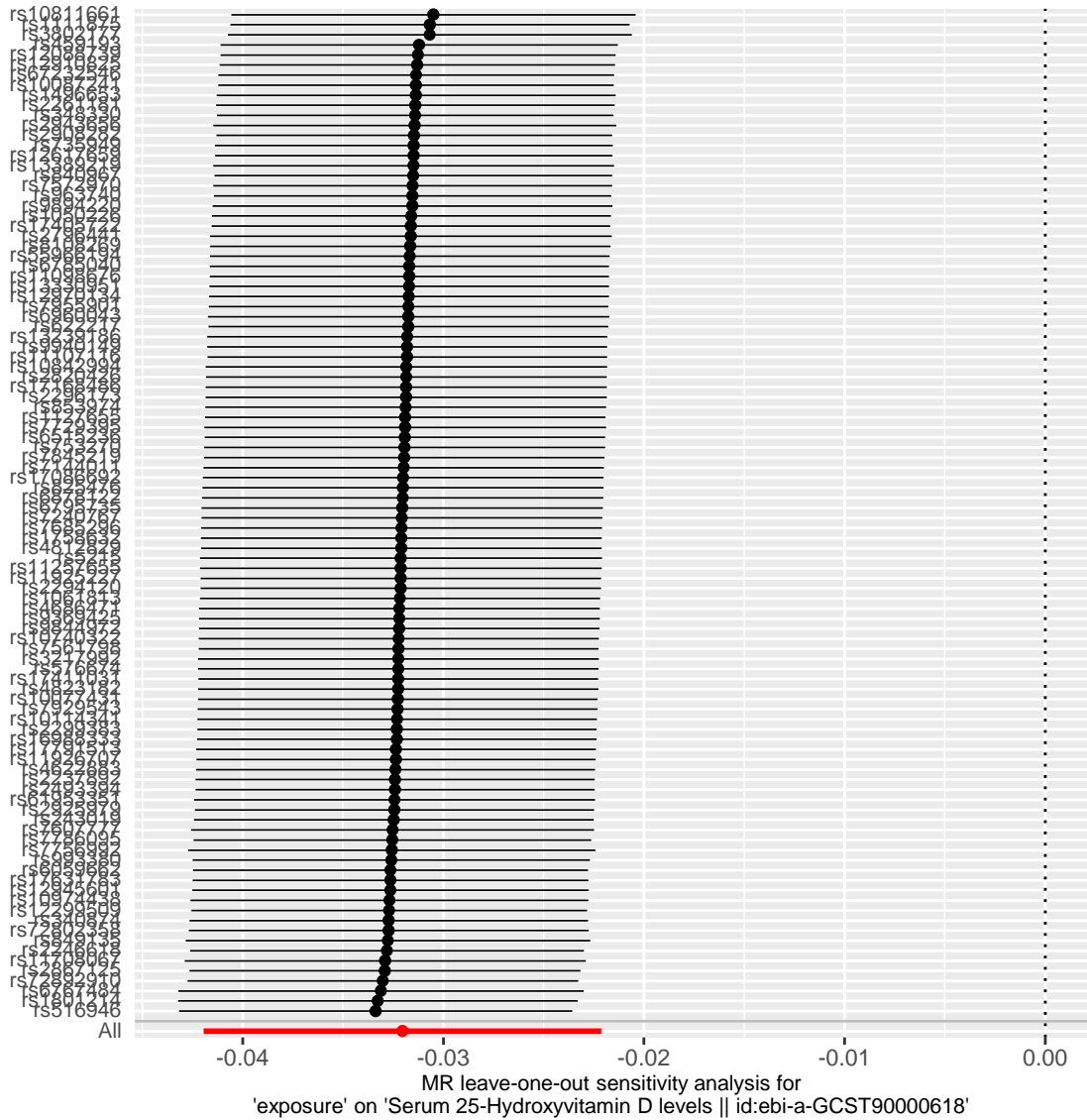

Supplement: Supplementary file 16 — Supplementary Material 16. [file 13098_2024_1383_MOESM16_ESM.pdf]

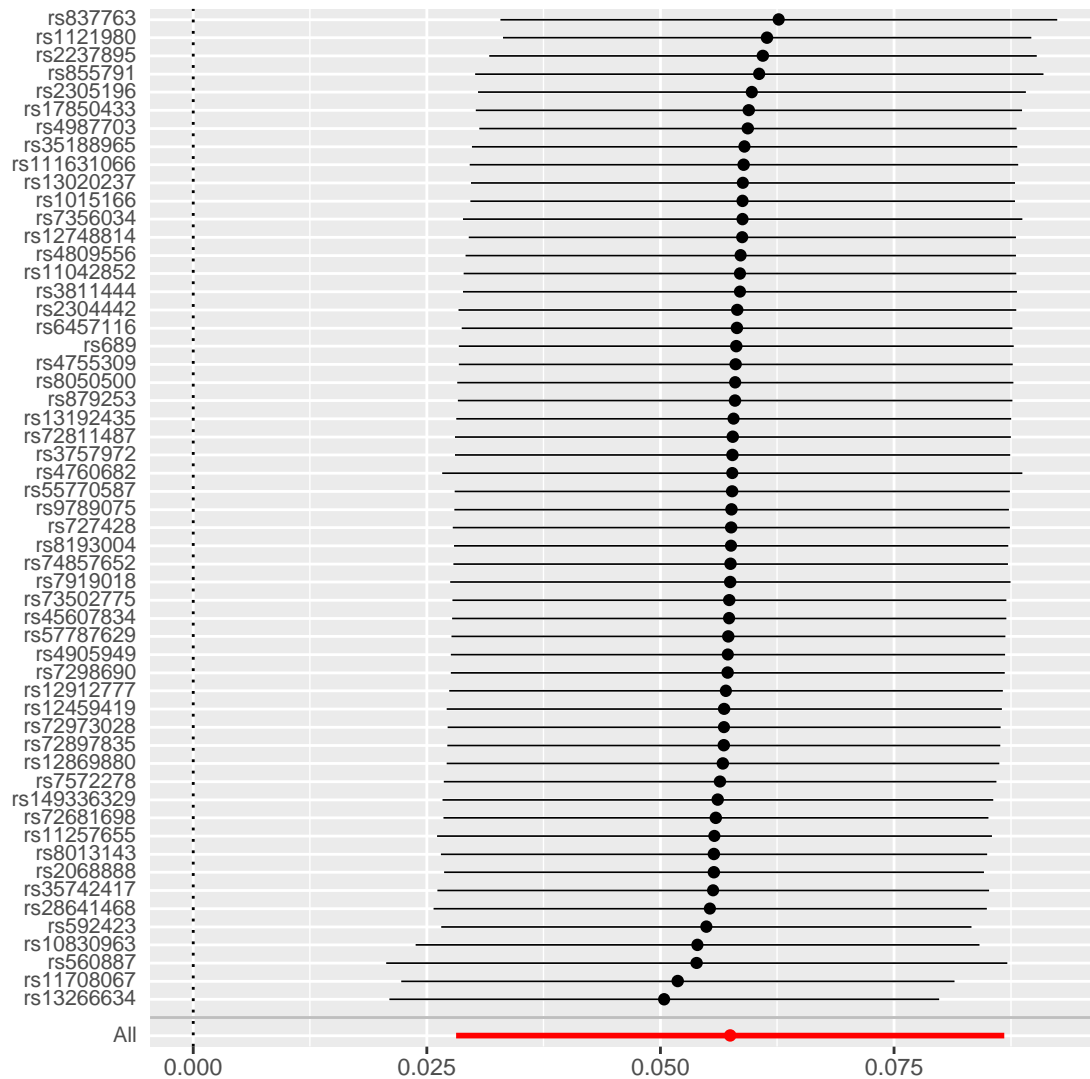

Supplement: Supplementary file 18 — Supplementary Material 18. [file 13098_2024_1383_MOESM18_ESM.pdf]

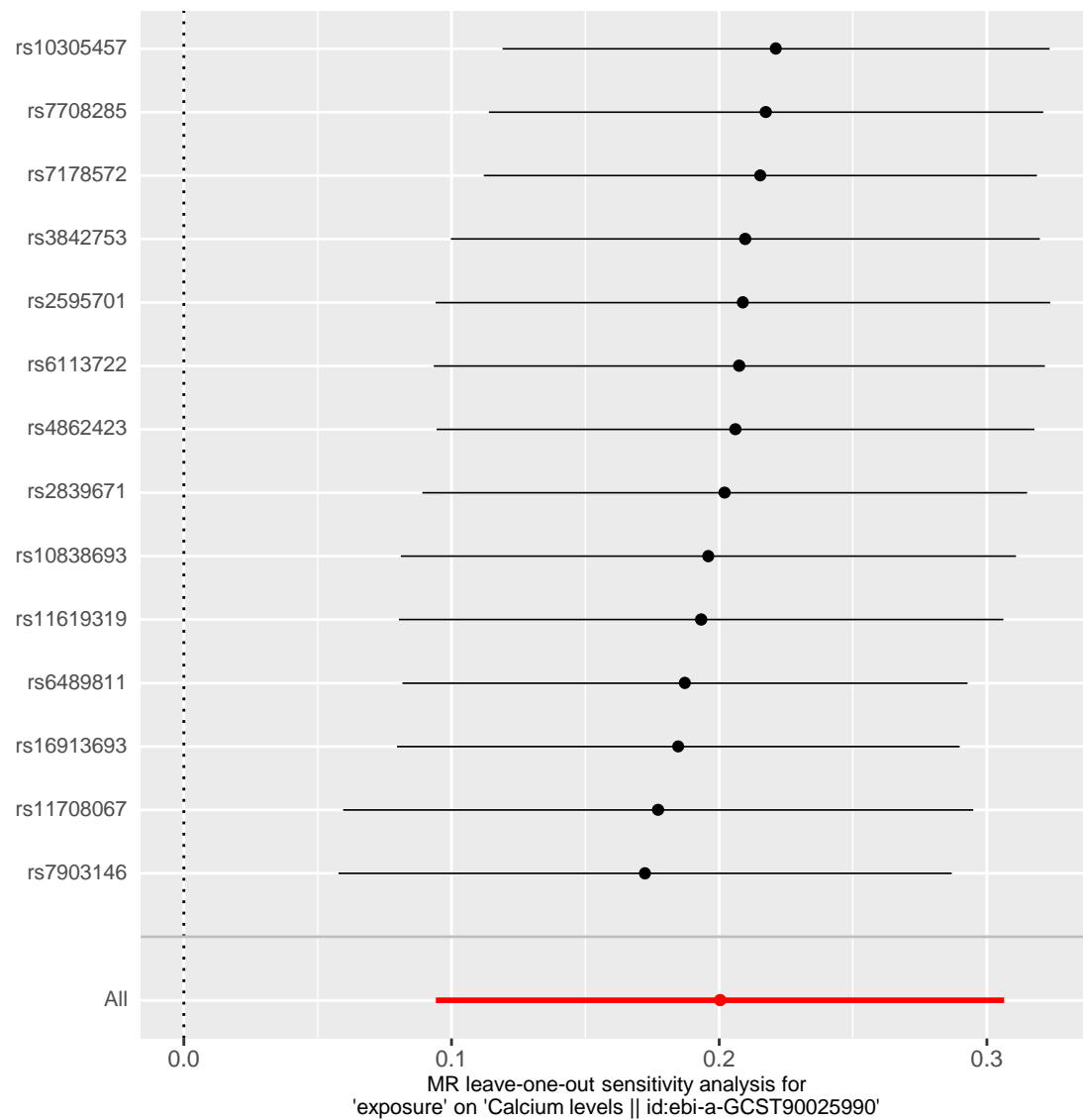

Supplement: Supplementary file 20 — Supplementary Material 20. [file 13098_2024_1383_MOESM20_ESM.pdf]

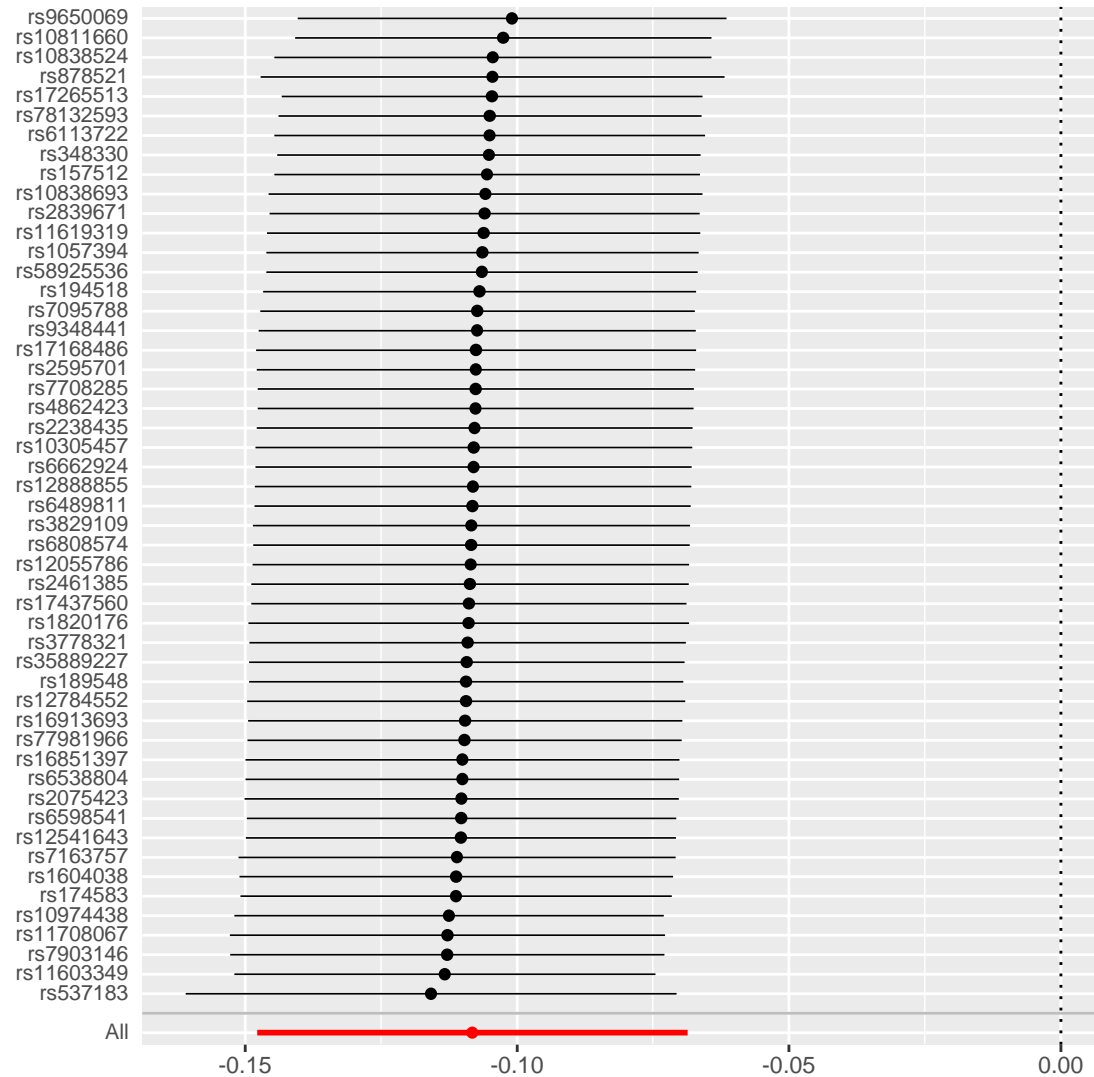

Supplement: Supplementary file 22 — Supplementary Material 22. [file 13098_2024_1383_MOESM22_ESM.pdf]

### MR Test

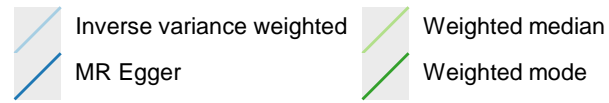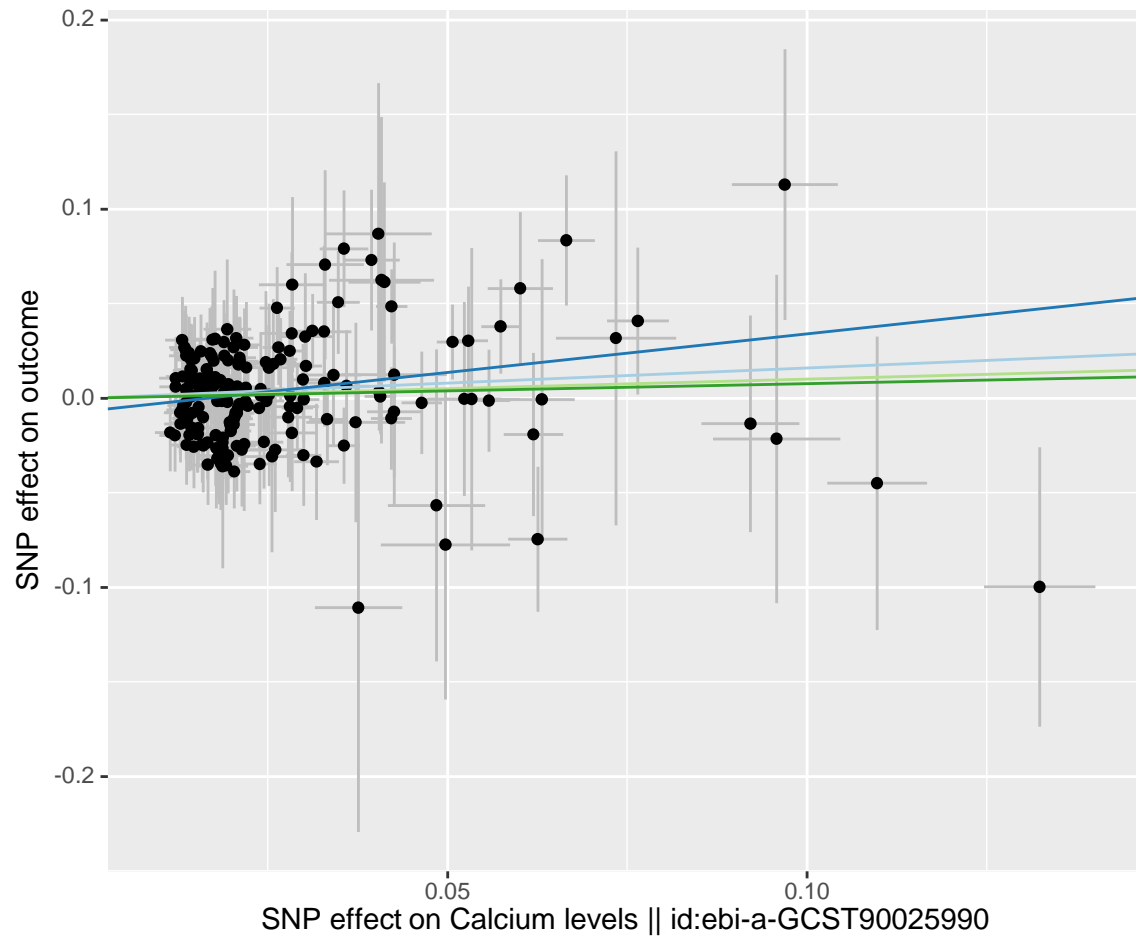

Supplement: Supplementary file 23 — Supplementary Material 23. [file 13098_2024_1383_MOESM23_ESM.pdf]

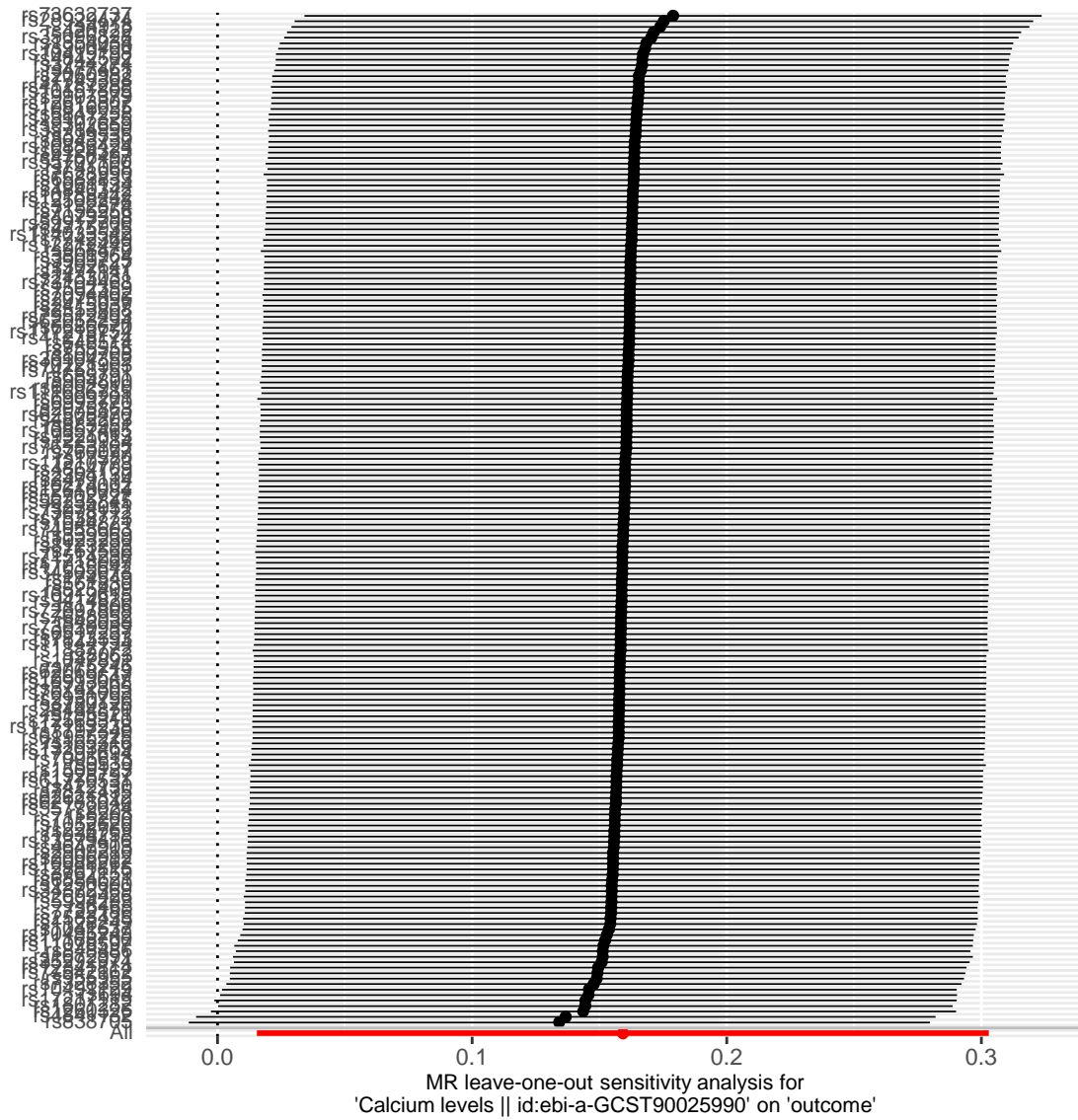

Supplement: Supplementary file 24 — Supplementary Material 24. [file 13098_2024_1383_MOESM24_ESM.pdf]

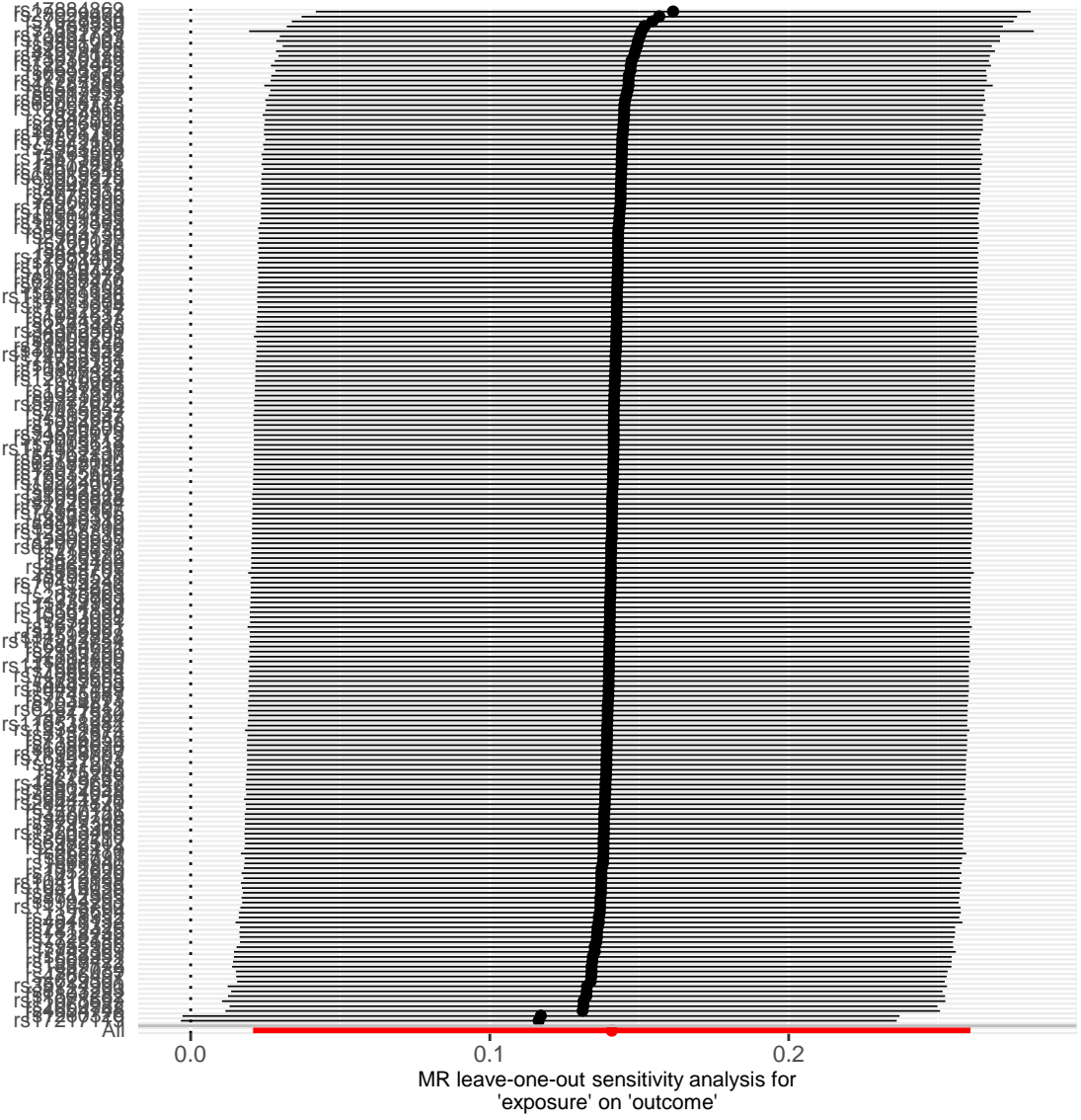

Supplement: Supplementary file 26 — Supplementary Material 26. [file 13098_2024_1383_MOESM26_ESM.pdf]
