# Supplementary material for: Exploring the mediating role of calcium homeostasis in the association between diabetes mellitus, glycemic traits, and vascular and valvular calcifications: a comprehensive Mendelian randomization analysis
Source: Diabetol Metab Syndr. 2024 Jun 22;16:136. doi: 10.1186/s13098-024-01383-z (PMC11193216; doi:10.1186/s13098-024-01383-z)
Supplement: Supplementary file 13 — Supplementary Material 13. [file 13098_2024_1383_MOESM13_ESM.pdf]

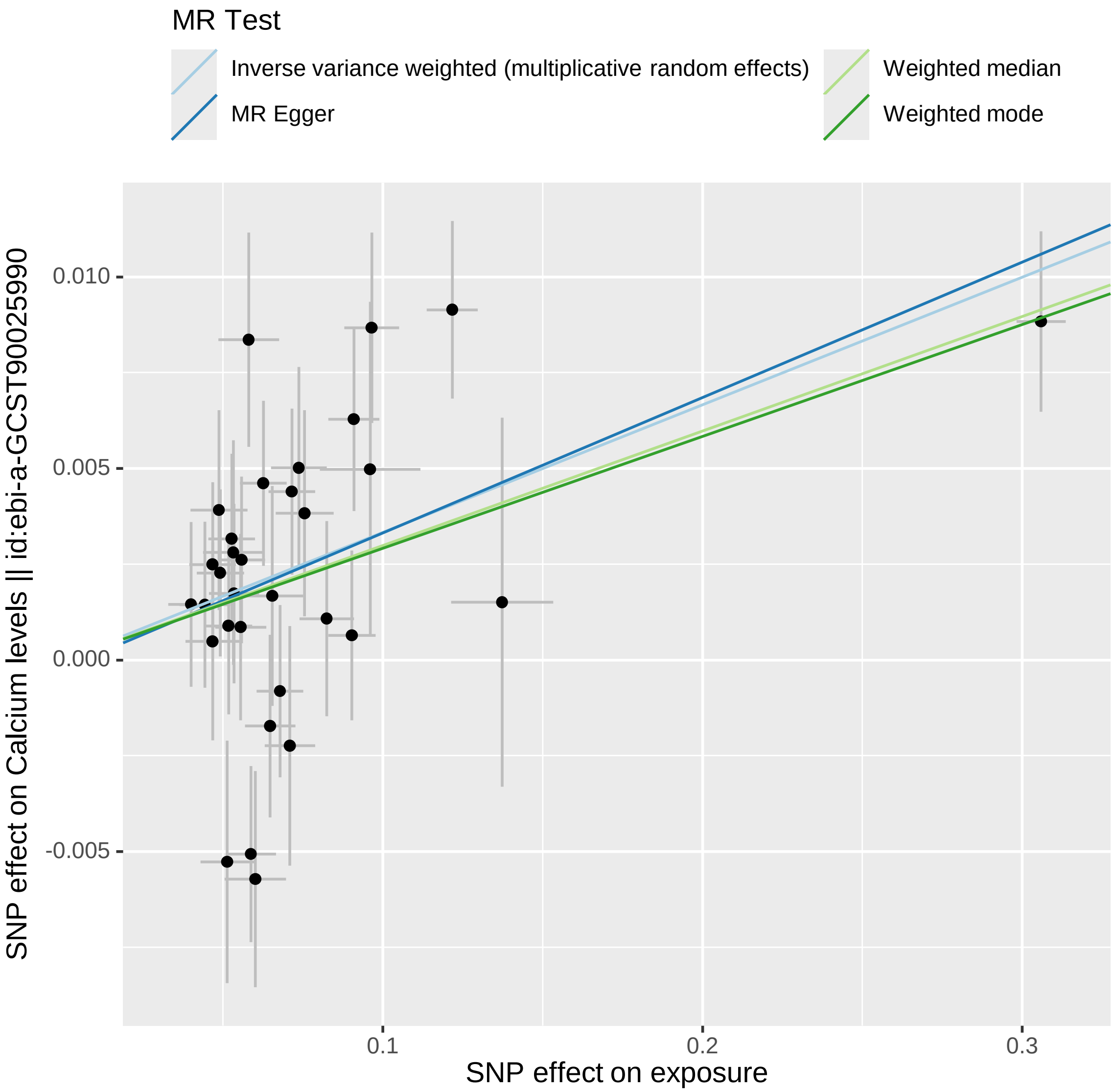
MR Test
Inverse variance weighted (multiplicative random effects)
MR Egger
Weighted median
Weighted mode
0.010
0.005
0.000
-0.005
SNP effect on Calcium levels || id:ebi-a-GCST90025990
0.1
0.2
0.3
SNP effect on exposure
