# Supplementary material for: Exploring the mediating role of calcium homeostasis in the association between diabetes mellitus, glycemic traits, and vascular and valvular calcifications: a comprehensive Mendelian randomization analysis
Source: Diabetol Metab Syndr. 2024 Jun 22;16:136. doi: 10.1186/s13098-024-01383-z (PMC11193216; doi:10.1186/s13098-024-01383-z)
Supplement: Supplementary file 21 — Supplementary Material 21. [file 13098_2024_1383_MOESM21_ESM.pdf]

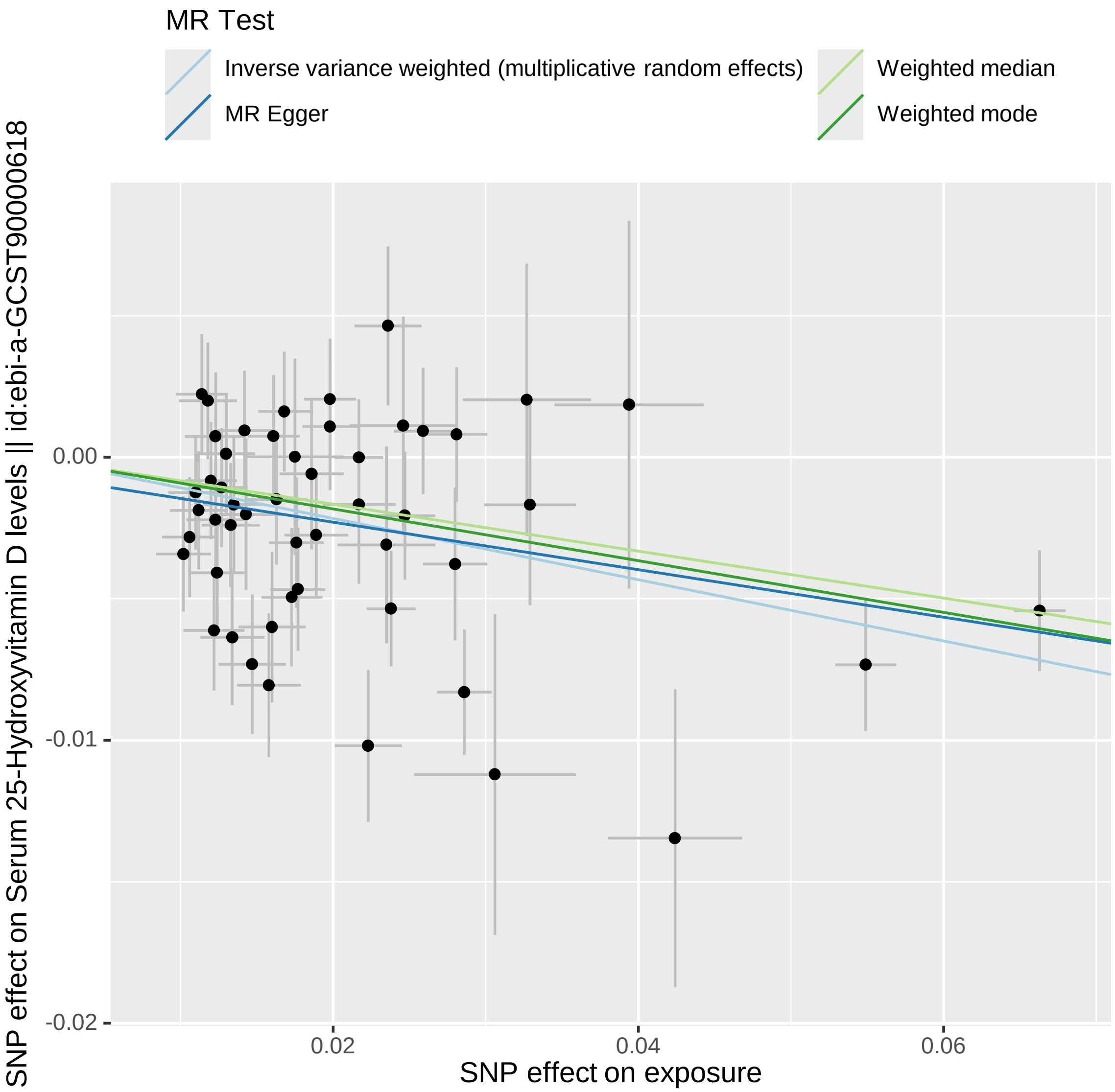
MR Test
Inverse variance weighted (multiplicative random effects)
MR Egger
Weighted median
Weighted mode
SNP effect on Serum 25-Hydroxyvitamin D levels || id:ebi-a-GCST90000618
0.00
-0.01
-0.02
0.02
0.04
0.06
SNP effect on exposure
