# Supplementary material for: Exploring the mediating role of calcium homeostasis in the association between diabetes mellitus, glycemic traits, and vascular and valvular calcifications: a comprehensive Mendelian randomization analysis
Source: Diabetol Metab Syndr. 2024 Jun 22;16:136. doi: 10.1186/s13098-024-01383-z (PMC11193216; doi:10.1186/s13098-024-01383-z)
Supplement: Supplementary file 27 — Supplementary Material 27. [file 13098_2024_1383_MOESM27_ESM.docx]

*Supplementary Figures*

Supplementary Figure S1 Scatter plots for T2DM on coronary artery calcification

Supplementary Figure S2 Leave-one-out analysis for T2DM on coronary artery calcification

Supplementary Figure S3 Scatter plots for T2DM on calcific aortic valvular stenosis

Supplementary Figure S4 Leave-one-out analysis for T2DM on calcific aortic valvular stenosis

Supplementary Figure S5 Scatter plots for HbA1c on coronary artery calcification

Supplementary Figure S6 Leave-one-out analysis for HbA1c on coronary artery calcification

Supplementary Figure S7 Scatter plots for HbA1c on calcific aortic valvular stenosis

Supplementary Figure S8 Leave-one-out analysis for HbA1c on calcific aortic valvular stenosis

Supplementary Figure S9 Scatter plots for FG on calcific aortic valvular stenosis

Supplementary Figure S10 Leave-one-out analysis for FG on calcific aortic valvular stenosis

Supplementary Figure S11 Scatter plots for calcific aortic valvular stenosis on FG

Supplementary Figure S12 Leave-one-out analysis for calcific aortic valvular stenosis on FG

Supplementary Figure S13 Scatter plots for T2DM on calcium levels

Supplementary Figure S14 Leave-one-out analysis for T2DM on calcium levels

Supplementary Figure S15 Scatter plots for T2DM on 25OHD

Supplementary Figure S16 Leave-one-out analysis for T2DM on 25OHD

Supplementary Figure S17 Scatter plots for HbA1c on calcium levels

Supplementary Figure S18 Leave-one-out analysis for HbA1c on calcium levels

Supplementary Figure S19 Scatter plots for FG on calcium levels

Supplementary Figure S20 Leave-one-out analysis for FG on calcium levels

Supplementary Figure S21 Scatter plots for FG on 25OHD

Supplementary Figure S22 Leave-one-out analysis for FG on 25OHD

Supplementary Figure S23 Scatter plots for calcium levels on coronary artery calcification

Supplementary Figure S24 Leave-one-out analysis for calcium levels on coronary artery calcification

Supplementary Figure S25 Scatter plots for calcium levels on calcific aortic valvular stenosis

Supplementary Figure S26 Leave-one-out analysis for calcium levels on calcific aortic valvular stenosis
